# SyFi: generating and using sequence fingerprints to distinguish SynCom isolates

**DOI:** 10.1099/mgen.0.001461

**Published:** 2025-09-04

**Authors:** Gijs Selten, Adrián Gómez-Repollés, Florian Lamouche, Simona Radutoiu, Ronnie de Jonge

**Affiliations:** 1 Department of Biology, Science for Life, Plant–Microbe Interactions, Utrecht University, Netherlands, 3584CH Utrecht; 2 Department of Molecular Biology and Genetics, Aarhus University, Aarhus, 8000C, Denmark; 3 Department of Information and Computing Sciences, Science for Life, AI Technology for Life, Utrecht University, 3584CC Utrecht, Netherlands

**Keywords:** amplicon sequencing, copy number variation, marker sequence, microbiome, synthetic community

## Abstract

The plant root microbiome is a complex community shaped by interactions among bacteria, the plant host and the environment. Synthetic community (SynCom) experiments help disentangle these interactions by inoculating host plants with a representative set of culturable microbial isolates from the natural root microbiome. Studying these simplified communities provides valuable insights into microbiome assembly and function. However, as SynComs become increasingly complex to better represent natural communities, bioinformatics challenges arise. Specifically, accurately identifying and quantifying SynCom members based on, for example, *16S rRNA* amplicon sequencing becomes more difficult due to the high similarity of the target amplicon, limiting downstream interpretations. Here, we present SynCom Fingerprinting (SyFi), a bioinformatics workflow designed to improve the resolution and accuracy of SynCom member identification. SyFi consists of three modules: the first module constructs a genomic fingerprint for each SynCom member based on its genome sequence and, when available, raw genomic reads, accounting for both copy number and sequence variation in the target gene. The second module extracts the target region from this genomic fingerprint to create a secondary fingerprint linked to the relevant amplicon sequence. The third module uses these fingerprints as a reference to perform pseudoalignment-based quantification of SynCom member abundance from amplicon sequencing reads. We demonstrate that SyFi outperforms standard amplicon analysis by leveraging natural intragenomic variation, enabling more precise differentiation of closely related SynCom members. As a result, SyFi enhances the reliability of microbiome experiments using complex SynComs, which more accurately reflect natural communities. This improved resolution is essential for advancing our understanding of the root microbiome and its impact on plant health and productivity in agricultural and ecological settings. SyFi is available at https://github.com/adriangeerre/SyFi.

**Impact Statement**

SynCom Fingerprinting (SyFi) represents a significant advancement in microbiome research by enhancing the accuracy and resolution of synthetic community (SynCom) member identification. By leveraging natural intragenomic variation, SyFi improves the differentiation of closely related microbial strains, addressing a key challenge in amplicon-based sequencing analysis. This increased precision allows researchers to more reliably track microbial dynamics in complex SynCom experiments, leading to deeper insights into microbiome assembly, function and host–microbe interactions. As a result, SyFi strengthens the interpretability of microbiome studies, ultimately contributing to a better understanding of plant health and productivity in both agricultural and ecological contexts.

## Data Summary

The data reported in this article have been deposited in the National Center for Biotechnology Information Short Read Archive BioProject database. SyFi fingerprint generation was run on a collection of 737 human gut-derived bacterial genomes from Forster *et al*. [1] (genomic read data deposited in the ENA under project numbers ERP105624 and ERP012217) and 447 Arabidopsis-derived bacterial genomes [2] [NCBI Project numbers PRJNA1138681, PRJNA1139421 (genomes) and PRJNA1131834 (genomic reads)]. A list of the closed genomes used for SyFi validation can be found at https://github.com/adriangeerre/SyFi.

Subsequently, SyFi was validated on a complex SynCom dataset by pseudoaligning *16S rRNA* V3-V4 and V5-V7 amplicon reads (PRJNA1191388) to SyFi-generated fingerprints and comparing this to shotgun metagenomics-sequenced dataset of the same samples in Selten *et al*. [3] (PRJNA1131994). This complex SynCom dataset included the inoculation of the 447 bacterial isolates on Arabidopsis, barley and lotus roots.

## Introduction

Sequencing the conserved *16S* rRNA gene (hereafter *16S rRNA*) is the benchmark method for microbiome community profiling across ecosystems [4,7]. The *16S rRNA* gene is found in all prokaryotes and consists of intermixed variable and conserved regions. The conserved regions facilitate the design of universal primer sequences compatible with diverse prokaryotes, enabling the amplification of variable regions. These variable regions are taxonomically informative, allowing for the identification, classification and quantification of prokaryotic micro-organisms within a community, thereby providing a broad overview of the microbiome composition across taxonomic levels [8]. Compared to shotgun metagenome sequencing, amplicon sequencing is cheaper, faster and applicable to host DNA-contaminated samples, though it has limitations [9]. Most notably, it is prone to PCR-induced amplification biases and has limited taxonomic resolution, often restricted to the genus level [9].

Adding another layer of complexity, bacterial strains can harbour multiple copies of the *16S rRNA* gene, which may differ in sequence [10,16]. Despite the fact that *16S rRNA* copy numbers can range from 1 to 15 copies per genome [17], they are often not accounted for in microbiome analyses [18]. Moreover, intragenomic *16S rRNA* copies can differ due to biological variations such as insertions, deletions or SNPs, making them difficult to distinguish from sequencing errors [14,19]. This inherent biological variation can distort bacterial abundance estimates, leading to a skewed representation of microbiome composition [14].

In recent years, synthetic community (SynCom) reconstitution experiments have emerged as a powerful approach in microbiome research [20,22]. In this bottom-up approach, hosts are inoculated with simplified yet representative microbial communities reconstituted from cultured bacterial isolates. SynCom experiments facilitate the investigation of host–microbe and microbe–microbe interactions and their effects on microbiome assembly and host function across taxonomic, molecular, genetic and functional levels. Currently, bioinformatic tools identify and quantify bacterial isolates in SynComs by matching amplicon sequence variants (ASVs) from microbiome datasets to *16S rRNA* amplicon sequences of SynCom members [23,24]. This approach is suitable for low-complexity SynComs, where inoculated members are phylogenetically distant and easily distinguishable by their genomic and amplicon sequences. However, as SynComs increase in complexity and include more closely related members, accurate taxonomic resolution becomes more challenging, which in turn complicates functional analyses. Additionally, current bioinformatic tools do not account for intragenomic *16S rRNA* copy number variation, further skewing bacterial abundance estimates.

To address these limitations, we developed SynCom Fingerprinting (SyFi), a bioinformatics workflow that identifies sequence variants and copy number variation of target microbial genes or gene regions in bacterial genomes. SyFi uses these gene variants as a reference fingerprint for pseudoalignment-based quantification, inspired by methods originally developed for transcript isoform quantification in transcriptomics [25,26]. We demonstrate that SyFi improves accuracy in identifying, distinguishing and quantifying SynCom members compared to current tools. Additionally, we compare microbiome compositions inferred by SyFi and other tools to metagenomic data, which serves as a gold standard. By improving the resolution of SynCom microbiome analyses, SyFi enables more complex SynCom studies, advancing our understanding of microbiome assembly and dynamics in plant-associated microbial communities.

## Methods

### SyFi workflow in brief

**Fig. 1.**
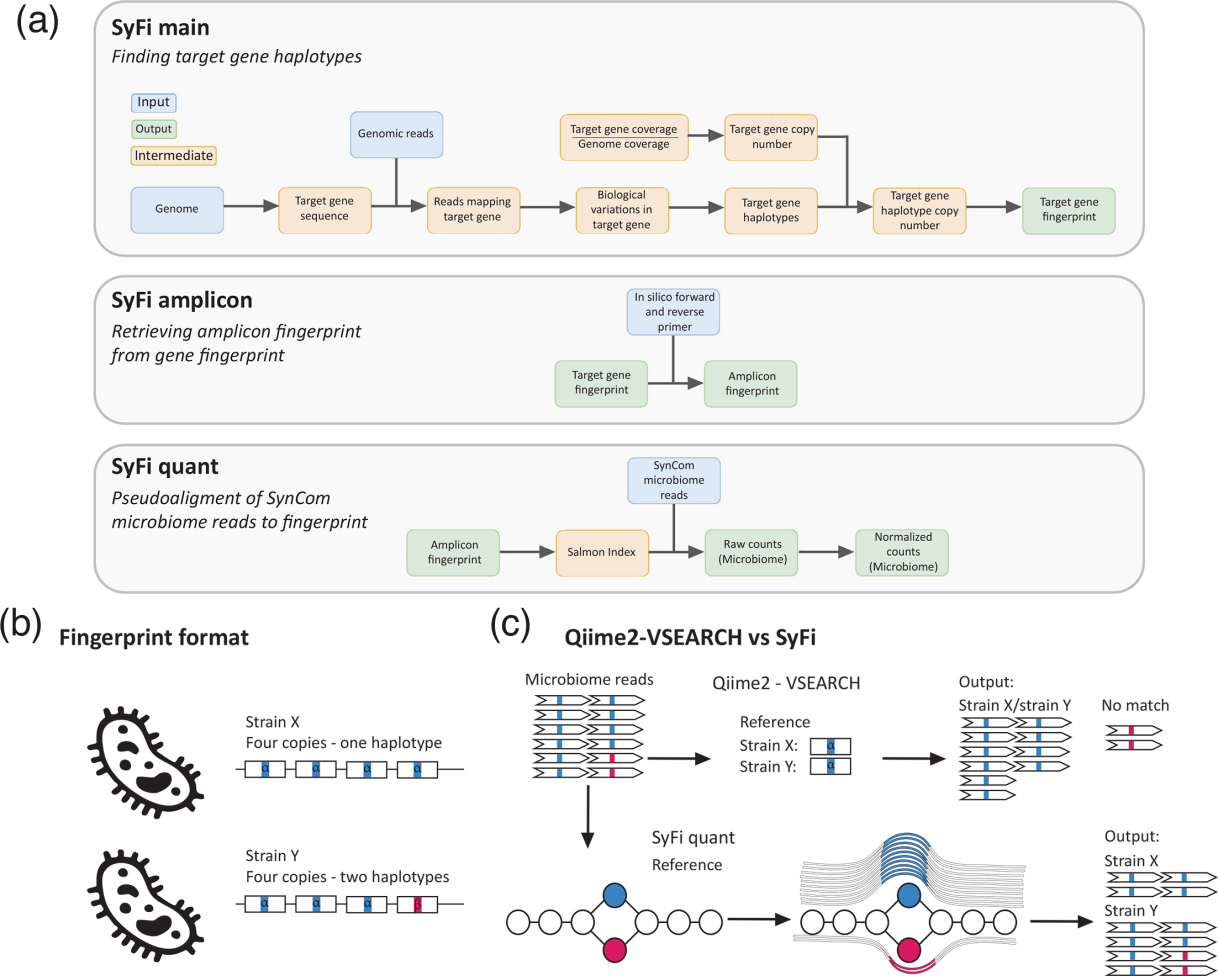
SyFi workflow and principle. (**a**) The SyFi pipeline consists of three modules. *SyFi main* generates a unique fingerprint for each SynCom member based on a reference target sequence. This involves extracting the gene sequence from the genome and isolating corresponding reads from genomic data. These reads are used to identify biological variations and construct haplotype variants of the target gene sequence. The final fingerprint is constructed using these haplotypes and their copy numbers. *SyFi amplicon* utilizes *in silico* primers to extract the amplicon region from the target gene fingerprint. *SyFi quant* indexes the (amplicon) fingerprints, pseudoaligns SynCom microbiome reads to this reference and normalizes raw counts based on the target gene’s copy number within the genomes. (**b**) A schematic overview of fingerprint sequences, where two near-isogenic strains share an identical target gene and copy number, except for one haplotype variant in strain Y containing a biological variation. (**c**) Benchmark tools typically extract a single reference sequence per strain, usually the most common haplotype. However, in near-isogenic strains, this approach may lead to indistinguishability. *SyFi quant* builds a De Bruijn graph *k*-mer index, pseudoaligns SynCom microbiome reads to the correct path within the graph and quantifies SynCom isolates. Small biological variations create splits in the De Bruijn graph, enabling higher resolution in distinguishing SynCom members.

The SyFi workflow consists of three modules. In the first module (*SyFi main*), SyFi builds a fingerprint of each SynCom member utilizing the genome sequence and the raw genomic reads used to build the genome as input (Fig. 1a-top and Appendix 1). Although without genomic reads, SyFi is unable to accurately call biological variations in the marker sequence, it is also possible to use available genome sequences directly. Aside from the genome sequence and genomic reads, SyFi requires a complete target sequence from which a fingerprint will be built. In microbiome studies, for example, the conserved bacterial *16S rRNA* sequence is commonly used for metagenomic amplicon sequencing and could therefore be used as the target sequence. Here, we focus our analysis and description on the use of SyFi to obtain *16S rRNA* fingerprints. However, other bacterial community markers, such as *recA* [27], *rpoB* [28] and *gyrB* [29] gene sequences, can also serve as input for SyFi.

Bacterial strains can harbour multiple copies of the *16S rRNA* gene [15], and these copies can have small biological variations like SNPs, insertions and deletions. SyFi is designed to identify all *16S rRNA* copies and biological variations in these *16S rRNA* sequences across a single bacterial genome. It aligns a representative sample *16S rRNA* sequence to the genome with the goal of retrieving the genomes’ *16S rRNA* sequence with the highest sequence identity score. Subsequently, SyFi uses raw genomic sequencing reads for read-based variant calling and phasing to determine within-target sequence biological variation and their co-occurrence. This information is used to assemble all unique *16S rRNA* sequences from the genome of interest, referred to as *16S rRNA* haplotypes. *SyFi main* calls variants with a minimum Phred-scaled mapping quality (MQ) confidence of 30 – corresponding to a 0.1% error rate. It also reports additional scores that assess strand bias, read MQ, base quality and bias related to the position of the variant in the sequence, which can be used to evaluate the haplotype quality. In the final step of the first module, SyFi determines the copy number of each *16S rRNA* haplotype by estimating the depth of coverage of each *16S rRNA* haplotype relative to the average genome-wide depth. Eventually, the sequences of all *16S rRNA* sequences from a given bacterial genome are concatenated into a fingerprint (Fig. 1b). A detailed overview of the first module can be found in Appendix 1.

The second module of SyFi (*SyFi amplicon*) allows the user to extract an amplicon fingerprint from the gene fingerprint using *in silico* primers (Fig. 1a-middle). Since the main SyFi module isn’t optimized for short target fragments, this module ensures the creation of fingerprints compatible with amplicon-sequenced SynCom datasets. In the third module (*SyFi quant*) (Fig. 1a-bottom, c), the fingerprint is used as a reference for pseudoalignment of metagenomic amplicon sequencing reads using Salmon (version 1.4.0) to accurately identify SynCom members and quantify their abundance (Fig. 1c) [26]. Salmon indexes the fingerprints using *k*-mers, creating a data structure (De Bruijn graph) to efficiently match sequencing reads. Pseudoalignment then assigns reads to compatible fingerprints by identifying shared *k*-mers, without performing full sequence alignment. Thus, the *SyFi quant* module reports the number of reads that are pseudoaligned to each SynCom member’s fingerprint and, in the final step, normalizes these counts based on the SyFi-predicted *16S rRNA* copy number to estimate SynCom member abundances. In the absence of genomic read files for *SyFi main*, it is also possible to extract marker haplotype sequences directly from the SynCom member’s genome by means of direct, blast-based sequence alignment, which can serve as a reference for *SyFi quant*. Similarly, marker sequences without known copy number variation, like *rpoB*, can skip *SyFi main* and serve directly as input for *SyFi quant* for quantification of SynCom members [30]. Additionally, *SyFi quant* reports average pseudoalignment confidence values for each SynCom member and sample (between 0 and 1), while also reporting the proportion of reads that are mapped to the SynCom member fingerprints.

The three SyFi modules differ in computational demands, with *SyFi main* requiring the most time and memory. Its memory usage is capped at 8 GB by default, though this can be increased if needed. Runtime is mainly determined by the size of the genomic read files used during haplotype identification (Fig. S1, available in the online Supplementary Material). In contrast, the performance of *SyFi amplicon* and *SyFi quant* largely depends on the tools they rely on: Qiime2 (version 2022.8) [31] and Salmon (version 1.4.0) [26], respectively. Users are advised to consult the documentation of these tools for detailed information on their system requirements.

### *16S rRNA* haplotype and copy numbers across bacterial taxa

To assess the prevalence of differential *16S rRNA* haplotypes in natural systems, we applied *SyFi main* to two large bacterial genomic datasets deriving from the human gut and plant root microbiomes [1,2]. *SyFi main* was run with default settings, using a full-length *16S rRNA* seed sequence (1,544 bp) from a *Paenisporosarcina* strain as the target (Data S1), which is approximately the proposed length of the *16S rRNA* sequence of 1,550 bp [8]. To maximize the recovery of complete *16S rRNA* genes across bacterial isolates, we allowed a maximum length deviation of 300 bp, accounting for natural variation in *16S rRNA gene* length.

### SyFi performance on a SynCom-inoculated root microbiome dataset

To assess the performance of SyFi with relation to SynCom member quantification in microbiome datasets, we made use of a complex SynCom dataset that included the bacterial genomes from the same Selten *et al*. [2] bacterial genome dataset. This dataset consists of 44 samples in which 3 different plant hosts were inoculated with a SynCom consisting of 447 different bacteria. The isolation, whole-genome sequencing, genome assembly of the bacteria and the experimental setup of the SynCom reconstitution experiment are described in [3].

To quantify the abundance of the 447 SynCom members in these 44 samples, we generated *16S rRNA* V3-V4 and V5-V7 amplicon sequencing data (Table S1) and performed untargeted shotgun metagenome sequencing [3] (Appendix 2). Pseudoalignment of amplicon reads to SyFi-generated fingerprints is most effective when the reference sequences correspond to the exact amplicon region covered by the reads [32] (Table S2). However, for accurate fingerprint generation, SyFi benefits from using the full-length target gene, as it requires a sufficient number of genomic reads mapping to the target gene to identify statistically supported biological variation. Therefore, we trimmed the already generated full-length *16S rRNA* fingerprints to V3-V4 and V5-V7 *16S rRNA* fingerprints using *SyFi amplicon* that utilizes the Qiime2’s read extraction algorithm (version 2022.8) [31]. To create *16S rRNA* fingerprints for the V3-V4 and V5-V7 regions, we used the following primers in *SyFi amplicon*: forward primer for V3-V4: CCTACGGGNGGCWGCAG, reverse primer for V3-V4: GACTACHVGGGTATCTAATCC, forward primer for V5-V7: AACMGGATTAGATACCCKG and reverse primer for V5-V7: ACGTCATCCCCACCTTCC [33].

### Quantification of SynCom members using the SyFi fingerprints

To quantify the SynCom members, we evaluated pseudoalignment of the amplicon reads to the V3-V4 and V5-V7 *16S rRNA* fingerprints using *SyFi quant*. The raw reads of the V3-V4 and V5-V7 *16S rRNA* amplicon datasets were filtered and denoised, and low-quality bases were trimmed at positions 20 and 240 at the 3′ end and 5′ end, respectively, for both forward and reverse reads using DADA2 (DADA2 version 1.26.0) [34]. The *16S rRNA* V3-V4 and V5-V7 amplicon data were pseudoaligned to the *16S rRNA* V3-V4 and V5-V7 fingerprints with Salmon (version 1.4.0) with default options except for a minimum mapping read identity score of 0.95 [26]. The number of pseudoaligned reads was subsequently normalized for *16S rRNA* copy number that was calculated in the first module.

### SyFi benchmarking tools

To benchmark SyFi’s accuracy in identifying, distinguishing and quantifying SynCom members from other tools, we also preprocessed the *16S rRNA* V3-V4 and V5-V7 datasets with Qiime2-VSEARCH, Mothur and Kraken2 [23,31, 35, 36]. Additionally, we performed direct pseudoalignment of microbiome amplicon reads to the original *16S rRNA* V3-V4 and V5-V7 amplicon sequences that we retrieved by a direct alignment to the genome. For this pseudoalignment, we used Salmon with the same parameters used in SyFi [26].

For Qiime2-VSEARCH, the microbiome amplicon reads were imported into Qiime2 and denoised by DADA2 as before (Qiime2 version 2022.11) [31]. The ASV sequences were matched with the reference V3-V4 or V5-V7 *16S rRNA* amplicon sequences using the Qiime2-VSEARCH plugin [23]. These references were retrieved from the *16S rRNA* sequence from SyFi’s first step [Fig. 1; blast alignment (v2.13.0)] with Qiime2 (version 2022.8) using the previously described V3-V4 and V5-V7 *16S rRNA* primers that were used in *SyFi amplicon*.

For Mothur (v.1.48.2), the microbiome amplicon reads were imported and built into contigs within the Mothur software [36]. Preprocessing the amplicon reads followed default options, which included filtering contigs with at least one ambiguous nucleotide, a length over 500 nt, and more than eight identical consecutive nucleotides. Furthermore, contigs were clustered based on a maximum of two nucleotide differences, counted and, similar to Qiime2-VSEARCH, matched with the original V3-V4 or V5-V7 *16S rRNA* sequences from SyFi’s first step. The query V3-V4 and V5-V7 *16S rRNA* sequences for Mothur were generated by an all-vs-all MAFFT alignment (v7.453) [37].

In the case of Kraken2 (version 2.1.3), a custom database was built from the original V3-V4 and V5-V7 *16S rRNA* sequences retrieved from SyFi’s first step or from the SyFi-generated V3-V4 and V5-V7 *16S rRNA* fingerprints [36]. The database was constructed using default options (*k*-mer length of 35 nt). Subsequently, Kraken2 was run with variable minimum hit groups (two, three and five) – indicating the minimum number of exactly matched *k*mers to the isolate to call it.

For all benchmark methods, including the Salmon pseudoalignment to amplicon sequences, we normalized absolute counts of the SynCom members by the *16S rRNA* copy number that *SyFi main* retrieved (Data S1–S3).

### Data analysis

To investigate SyFi’s ability to distinguish isolates based on *16S rRNA*, V3-V4 or V5-V7 fingerprints, we clustered the fingerprints at 100% sequence similarity with VSEARCH (VSEARCH version 1.2.13) [23]. The established clusters of isolates were subsequently used to compare the bacterial abundances in the fingerprint dataset with the abundances in the metagenome-sequenced dataset. The abundances of isolates of the same cluster were summed in both the fingerprint and metagenome datasets to ascertain a fair comparison of identical isolates. Afterwards, the abundances were normalized to relative counts, and isolates that were absent in either dataset were removed in both the fingerprint and metagenome datasets.

SyFi’s accuracy in quantifying the SynCom members was assessed by calculating the normalized root mean deviation score (normalized root mean square error, NRMSE) (Formula A) – a value that assesses the error between the fingerprint dataset (*a*) and the metagenome-sequenced (*m*) dataset. The error is calculated across bacterial genera, species or SynCom members (*i*) and summed (*K*) to calculate the deviation. To assess this value for the genera, we collapsed the SynCom member abundances by genome taxonomy database-inferred genera (version v2.3.0) [38]. To ascertain the NRMSE value for the bacterial species, we collapsed the SynCom members into clusters based on 95% genome sequence identity with dRep (version 3.4.1) [39].


$$\text{A) }\;\text{NRMSE}=\sqrt{\frac{1}{K}\sum_{i=1}^{K}\frac{(a_{i}-m_{i}{)}^{2}}{\frac{a_{i}+m_{i}}{2}}}$$

SyFi’s accuracy in identifying the presence/absence of genera, species and SynCom members included a presence/absence threshold for both the fingerprint and metagenomics-sequenced datasets. Strains with a minimum relative abundance of 0.5% were assigned as being present. Performance was assessed by accuracy (proportion of isolates correctly identified as present or absent), precision (proportion of identified present isolates that are indeed present), recall or taxon sensitivity (proportion of the present isolates correctly identified as present) and taxon specificity (proportion of identified absent isolates that are indeed absent). For the identification, the metagenome-sequenced dataset was subsetted for the isolates with fingerprints to allow a fair comparison.

All data analysis steps described above were performed identically for the Qiime2-VSEARCH, Mothur and Kraken2 analyses of the SynCom dataset, as well as the Salmon run on the original amplicon sequences. The quantification for the benchmark tools was assessed with the amplicon sequences retrieved from SyFi’s first alignment step. For the identification, the datasets were similarly subsetted to only include the isolates for which SyFi retrieved fingerprints.

## Results

### The principle of SyFi

SyFi is inspired and partially built upon the principle of pseudoalignment-based quantification methods used in transcriptomics. This approach involves creating a *k*mer-based reference for near-isogenic gene isoforms, which can be distinguished through pseudoalignment. In this method, reads are matched to the correct isoform by aligning with the appropriate *k*-mer paths in a De Bruijn graph (Fig. 1c). Minor biological variations in a near-isogenic bacterial strain’s gene reference cause splits in these *k*-mer paths, enabling the accurate identification of the correct isoform or SynCom member, even when they are highly similar.

SyFi consists of three modules. The first module, *SyFi main* (Fig. 1a-top and Appendix 1), constructs a genomic fingerprint for each micro-organism to be quantified later. *SyFi main* uses the genome and genomic reads of bacterial strains to identify variants of a target sequence and builds a fingerprint consisting of a concatenation of all detected gene variants and/or copies, here illustrated for the *16S rRNA gene*. Target sequence copies can be identical but can also differ in their sequence, frequently referred to as haplotypes, due to SNPs, insertions or deletions (Fig. 1b). The second module, *SyFi amplicon* (Fig. 1a-middle), extracts regions from the fingerprints, essential for amplicon sequence analysis. Such analysis usually focuses on a smaller region of the target gene, for example, a targeted variable region (or regions) of the *16S rRNA* gene that can be captured by short-read sequencing. Finally, the third module, *SyFi quant*, quantifies microbial abundance by leveraging biological variation between amplicon sequences (Fig. 1a-bottom, c). This is achieved by pseudoalignment-based quantification, implemented in Salmon [26]. By distinguishing highly similar fingerprints from closely related microbes, this approach enables more accurate quantification, analogous to the analysis of alternative splicing in transcriptomics [40] (Fig. 1c).

### SyFi distinguishes more bacteria

To assess whether SyFi’s ability to distinguish bacteria is more effective than standard methods, we evaluated *SyFi main*’s performance on two large bacterial genomic datasets [1,2] and *SyFi quant*’s performance on a complex SynCom dataset [3]. SyFi was applied to genome sequences of 710 human gut- and 447 *Arabidopsis thaliana* (hereafter Arabidopsis) root-derived bacterial isolates, successfully retrieving *16S rRNA* fingerprints for 458 and 382 isolates, respectively (Table 1). The isolates for which SyFi was unable to construct a fingerprint either lacked the *16S rRNA* gene or had an incomplete gene sequence.

**Table 1.** Comparison of SyFi-generated *16S rRNA* fingerprints and directly retrieved *16S rRNA* sequences in distinguishing bacterial isolates The first column indicates whether VSEARCH was applied to the original *16S rRNA* sequences extracted directly from the bacterial genomes, clustered at varying identity thresholds (95, 97, 98, 99 and 100%), or to the SyFi-generated fingerprints clustered at 100% sequence identity (SyFi – 100).

	VSEARCH clustering percentage (%)	No. of clusters	No. of singletons	No. of isolates in largest cluster	Average no. of isolates in cluster	No. of isolates
Human gut	**95**	140	58	31	4	**567**
	**97**	191	88	22	3	**567**
	**98**	217	102	17	2.6	**567**
	**99**	255	135	14	2.2	**567**
	**100**	423	343	8	1.3	**567**
	**SyFi – 100**	**451**	**446**	**3**	**1**	458
Plant root	**95**	64	15	40	6.5	**417**
	**97**	88	34	36	4.7	**417**
	**98**	108	47	30	3.9	**417**
	**99**	141	73	29	3.0	**417**
	**100**	210	146	22	2.0	**417**
	**SyFi – 100**	**326**	**300**	**8**	**1.2**	378*

*Although 382 *16S rRNA* fingerprints were obtained, 4 sequences were excluded due to exceeding VSEARCH’s maximum sequence length threshold.

To determine whether genome assembly fragmentation contributed to the missing *16S rRNA* sequences, we compared genome completeness, coverage and L50 – three indicators of genome assembly integrity – between isolates with and without SyFi-generated *16S rRNA* fingerprints for the plant root bacterial genome dataset (Fig. S2). Isolates with SyFi-identified fingerprints had significantly higher genome completeness and coverage, while those without fingerprints exhibited greater genome fragmentation (low L50) and a higher number of scaffolds. For the user, it is therefore important to assess the genome assembly quality as incomplete or fragmented genomes reduce SyFi’s ability to retrieve *16S rRNA* fingerprints.

We next assessed the discriminative power of SyFi-generated *16S rRNA* fingerprints using VSEARCH clustering at 100% sequence identity (Table 1). This analysis identified 451 and 326 non-redundant *16S rRNA* fingerprints for the human gut and plant root datasets, respectively, with 446 and 300 being unique. In comparison, direct alignment-based clustering retrieved a higher number of *16S rRNA* sequences – 567 for the human gut and 417 for the plant root dataset – but these included partial and fragmented sequences. In spite of that difference, we found only 423 and 210 non-redundant sequences for the human gut and plant root datasets, with just 343 and 146 being unique, respectively. Overall, SyFi improved isolate resolution across both datasets, distinguishing 98.5% (451/458) of human gut isolates and 86.2% (326/378) of plant root isolates. This was a substantial improvement over Qiime2-VSEARCH, which identified only 74.6% (423/567) and 47.0% (210/417) of isolates, respectively. Furthermore, SyFi outperformed Qiime2-VSEARCH not only at the full-length *16S rRNA* level but also within the V3-V4 and V5-V7 amplicon regions (Table S3).

### SyFi identifies a similar *16S rRNA* copy number distribution across the bacterial kingdom

**Fig. 2.**
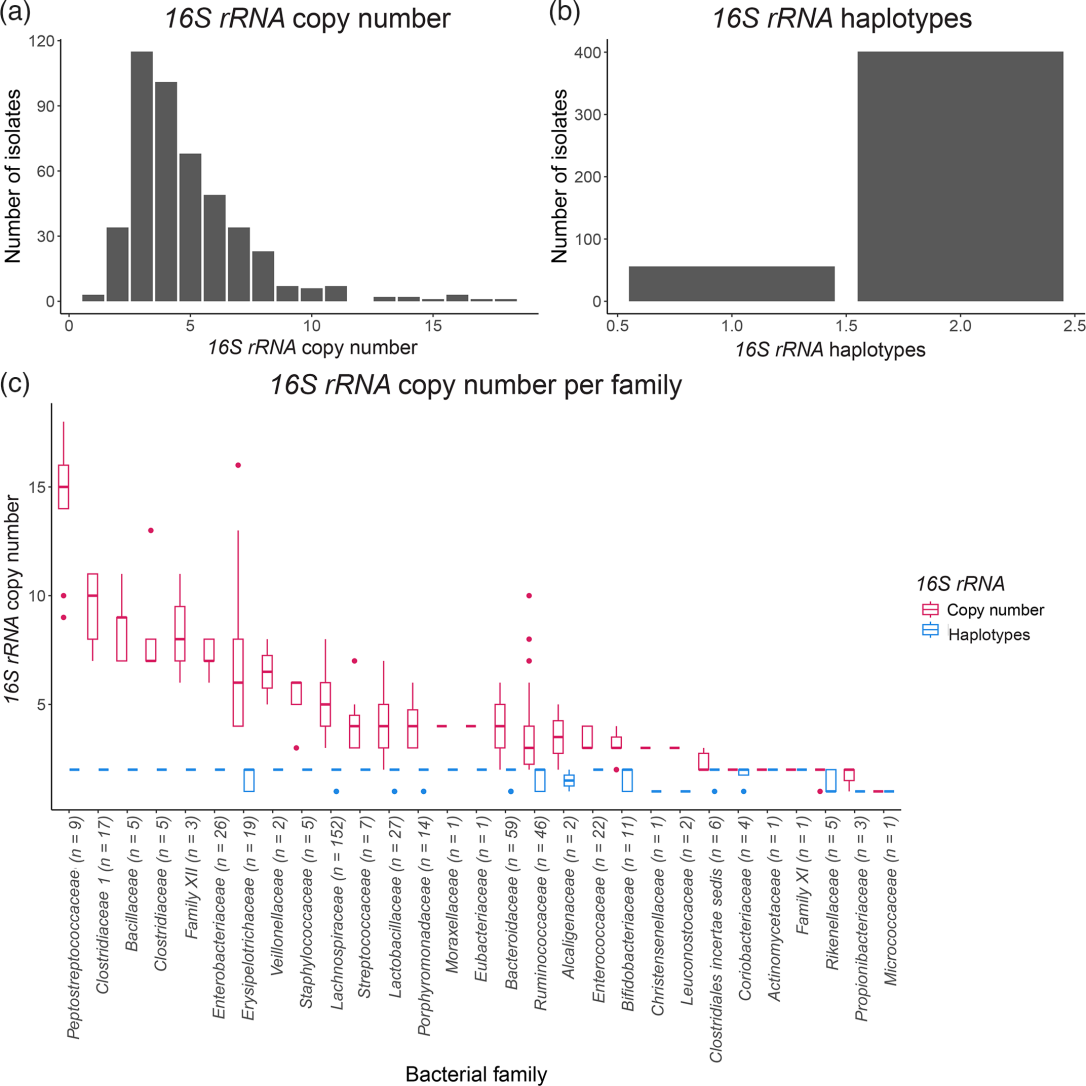
SyFi-derived *16S rRNA* copy number and haplotype distribution among 458 human gut bacterial isolates. (**a**) *16S rRNA* copy number. (**b**) *16S rRNA* haplotype distribution. (**c**) Distribution of *16S rRNA* copy number (red) and the number of *16S rRNA* haplotypes (turquoise) within diverse bacterial families. Families are ordered by the average number of *16S rRNA* copies in descending order. The number in brackets indicates the number of isolates in the respective family.

For both the human gut and the plant root, SyFi identified most bacterial genomes as having two to five *16S rRNA* copies (Figs2a  3a), with the majority containing at least two distinct haplotypes (Figs2b  3b), a feat especially present in the human gut microbiome. These findings align with previous analyses of over 2,000 bacterial genomes [15,16]. Standard direct alignment methods, by contrast, typically detect only a single *16S rRNA* haplotype per genome (Fig. S3), illustrating how conventional approaches often overlook intragenomic variation.

**Fig. 3.**
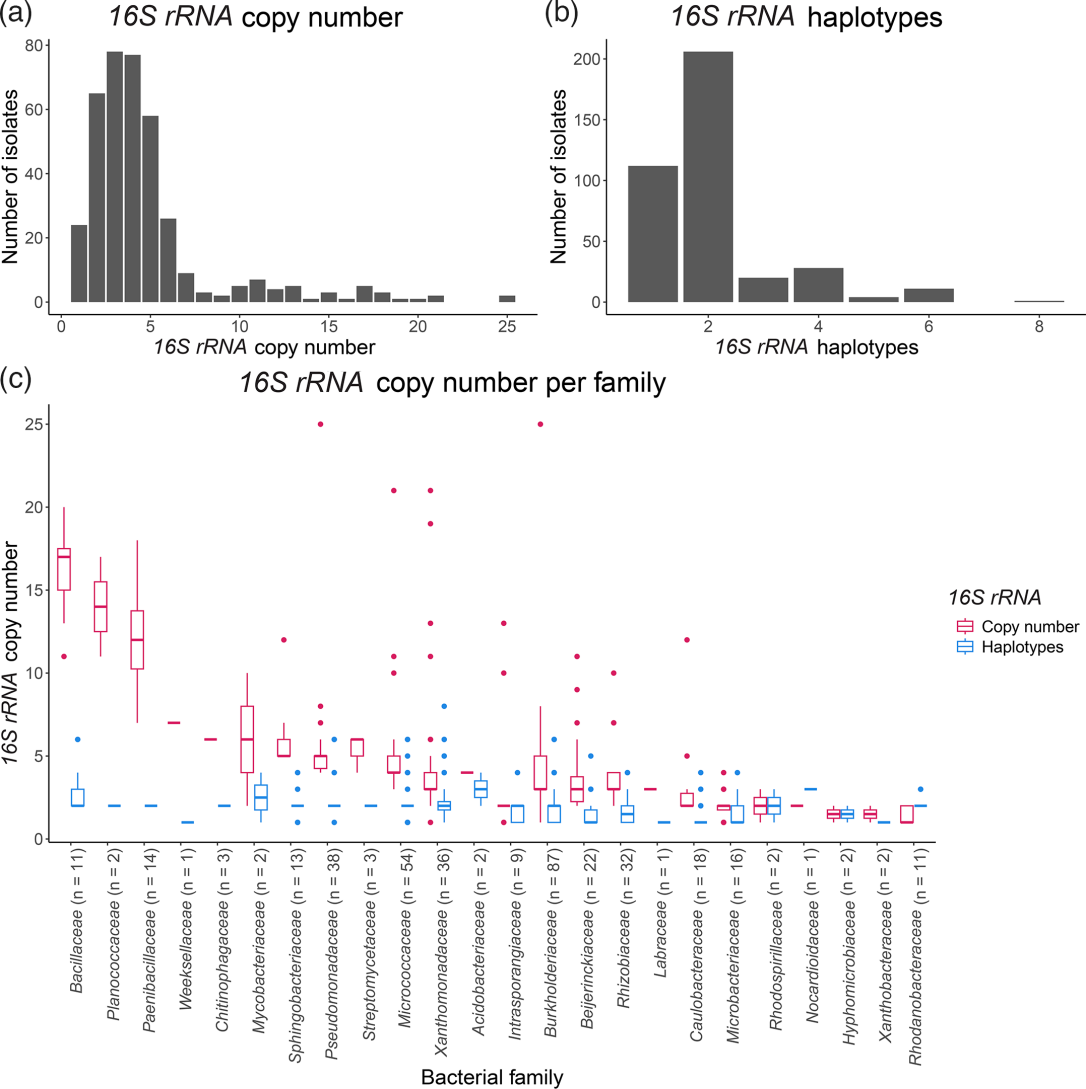
SyFi-derived *16S rRNA* copy number and haplotype distribution among 382 bacterial isolates. (**a**) *16S rRNA* copy number. (**b**) *16S rRNA* haplotype distribution. (**c**) Distribution of *16S rRNA* copy number (red) and the number of *16S rRNA* haplotypes (turquoise) within diverse bacterial families. Families are ordered by the average number of *16S rRNA* copies in descending order. The number in brackets indicates the number of isolates in the respective family.

To validate these observations with recent data, we analysed 3,615 complete bacterial genomes retrieved from National Center for Biotechnology Information (NCBI) (15 April 2024), spanning the entire bacterial kingdom (Fig. S4A, B). Consistent with prior studies, we found that most genomes contain two to five *16S rRNA* copies (90.7%) but only one detected haplotype [15,16]. Additionally, across all datasets, the bacterial families *Bacillaceae*, *Planococcaceae* and *Paenibacillaceae* had the highest *16S rRNA* copy numbers (Figs2c 3c and S4C, D), reinforcing SyFi’s accuracy in estimating *16S rRNA* copy number.

The prevalence of only one detected haplotype in complete bacterial genomes is surprising. Bacterial genome assembly often relies on Illumina short-read sequencing combined with PacBio or Nanopore long-read sequencing [41]. However, these methods may interpret true sequence variation as sequencing errors and erroneously correct them, potentially explaining the limited haplotype diversity observed. Although genome-polishing tools are improving [42], many complete genome assemblies remain unpolished. To investigate whether there is an underestimation of the number of *16S rRNA* haplotypes in the complete genomes, we analysed the *16S rRNA* gene(s) in ten *Bacillales* strains with complete genomes and for which raw Illumina short reads were available. blastn alignment of the *16S rRNA* gene (Sequence S1) to these ten genomes, indeed, yielded a single *16S rRNA* haplotype for nine out of ten strains. However, alignment of the corresponding short-read sequences by SyFi yielded two distinct haplotypes in all ten cases (Table S4) with clear biological variations at specific positions in the *16S rRNA* sequence (Fig. S5). This highlights an important limitation in genome assembly, where complete genomes potentially lack biological variation, whereas SyFi can recover this variation, providing a more accurate overview of sequence diversity.

While complete genomes may underestimate *16S rRNA* haplotype diversity, fragmented or draft genome assemblies are more prone to contamination, which can compromise the accuracy of *16S rRNA* fingerprint construction. A detailed analysis of sequence contamination, genomic heterogeneity and G+C content showed that SyFi may overestimate *16S rRNA* copy numbers under conditions of moderate (>5%) to high (>10%) contamination, increased heterogeneity (>25%) and elevated G+C content (>0.85 G+C ratio *16S rRNA*:genome) (Appendix 3). Therefore, we strongly recommend that users assess the quality of genome assemblies and apply genome-cleaning tools to remove potential contaminants prior to SyFi analysis. Although SyFi currently has limited capacity to correct for G+C content effects (Appendix 3), future versions may incorporate such adjustments.

### SyFi outperforms VSEARCH in identifying and quantifying bacterial isolates

To assess SyFi’s accuracy in profiling SynCom-inoculated microbiome samples, we analysed a complex SynCom dataset comprising 447 bacterial isolates inoculated on plant roots [3]. We sequenced the V3-V4 and V5-V7 *16S rRNA* amplicons from the root microbiome samples and pseudoaligned the amplicon reads to SyFi-generated *16S rRNA* fingerprints using *SyFi quant*. First, we optimized pseudoalignment accuracy by testing various minimum sequence similarity thresholds in Salmon while maximizing the number of pseudoaligned reads (Figs S6 and S7). We then compared SyFi-based quantifications to metagenome sequencing – considered the gold standard for validating amplicon sequencing datasets [43,45] – by computing the NRMSE. A low NRMSE value indicates a smaller deviation from the metagenome-sequenced dataset and thus a higher weighted accuracy.

**Fig. 4.**
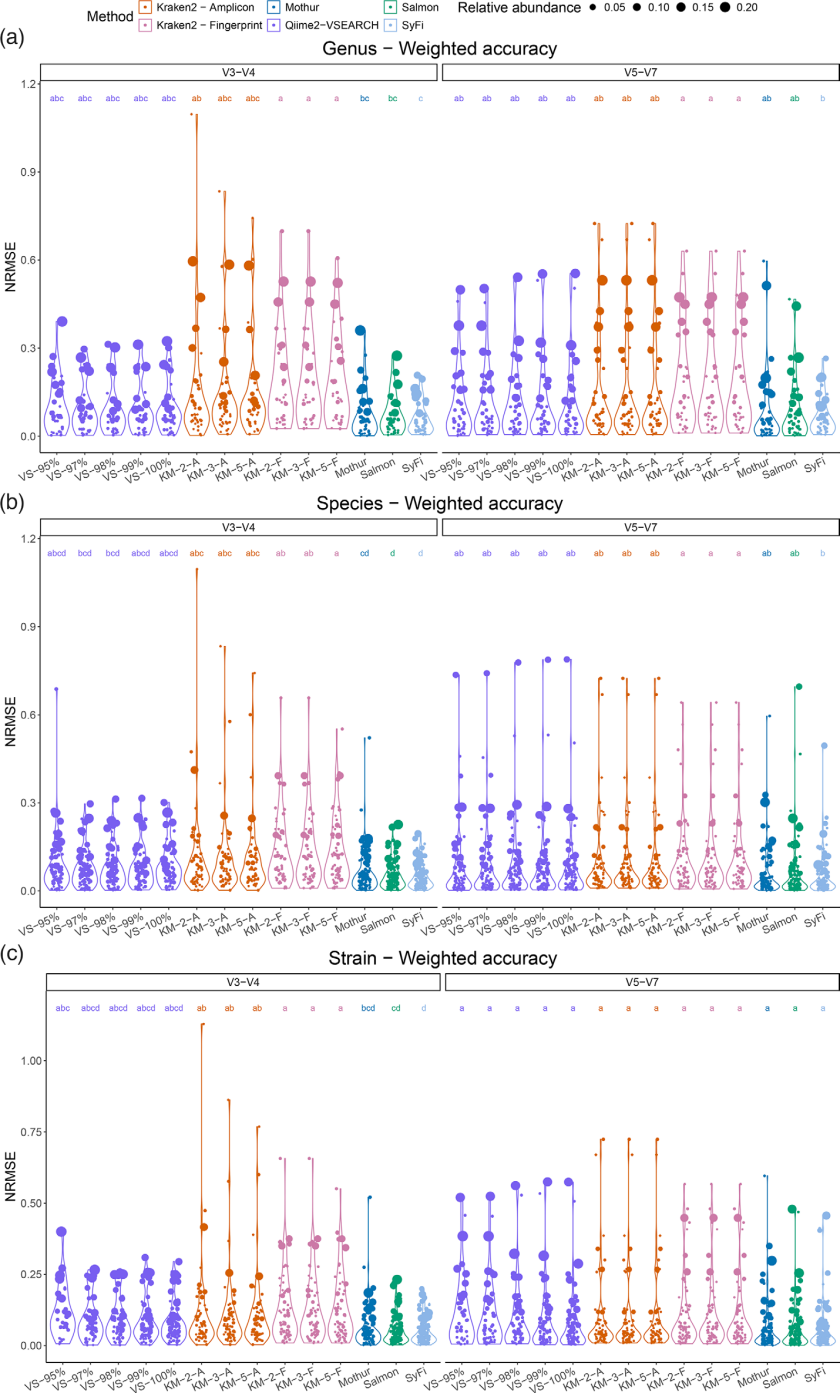
Weighted accuracy of SyFi in comparison with benchmark tools. For each (a) bacterial genus, (b) species and (c) strain, the error to the metagenome-sequenced dataset is computed as the NRMSE value (*y*-axis). Each coordinate indicates a bacterial genus, species or strain, whose relative abundance in the metagenome-sequenced dataset is indicated by the size. The *x*-axis refers to the tool applied on the data, with mapping sequence identity percentages for Qiime2-VSEARCH (VS) and number of *k*mer-matches for kraken2 (KM) using either the amplicon sequences (A) or SyFi-generated fingerprints (F) as reference. Statistical differences are indicated by ANOVAs and post-hoc tests (*P*<0.05).

Indicatively, the NRMSE values from genus, species and strain quantification are the lowest for SyFi, followed by direct pseudoalignment to the amplicon reads with Salmon and Mothur (Figs4  5). For individual genera, species and strains, however, these three methodologies are not statistically different (Fig. 4), while they are in comparison to Kraken2. Also, bacterial genera, species or strains that have a moderate to high relative abundance are quantified most accurately by SyFi, followed by Mothur, while Qiime2-VSEARCH displays reduced accuracy in the quantification of these groups (Fig. 4).

**Fig. 5.**
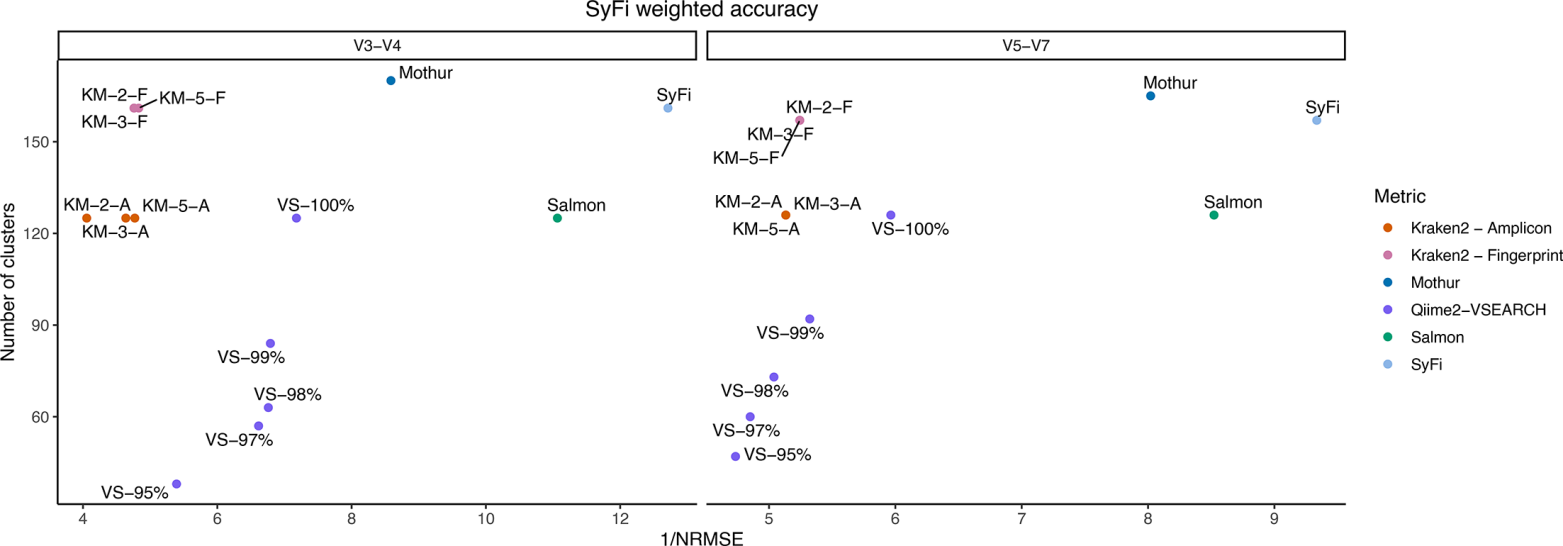
SyFi weighted accuracy. On the *y*-axis, *SyFi main*’s enhanced performance is indicated by the number of strains (or clusters) that it is able to distinguish (*y*-axis) in comparison with other benchmark tools. On the *x*-axis, *SyFi quant*’s enhanced performance is indicated by the inverse of the NRMSE value – the error to the metagenomics-sequenced dataset. The higher the inverse NRMSE value, the more similar the data is to the metagenome-sequenced dataset. The inverse NRMSE values are computed from averaging the individual NRMSE values per bacterial strain and calculating the inverse. Benchmark tools Qiime2-VSEARCH and Kraken2 were run with multiple mapping sequence identity percentages (VS) and number of *k*mer-matches (KM), respectively. For Kraken2, either the amplicon sequences (A) or SyFi-generated fingerprints (F) were used as reference.

In terms of correct identification of bacterial genera, species and strains’ presence or absence, SyFi performs consistently well, although it does not outperform other tools in a similar consistent manner (Table S5). SyFi accurately identifies genera, species and strains in the *16S rRNA* V3-V4 amplicon data, while Qiime2-VSEARCH at 98 or 99% sequence identity matching is most accurate for the *16S rRNA* V5-V7 amplicon data. Despite this result, it is important to note that SyFi can identify more strains correctly (Fig. 5), thus corroborating its high resolution in identifying the correct strain in the data.

Conclusively, SyFi allows the identification of more isolates (Fig. 5) and enhances accuracy in the quantification of bacterial genera, species and strains (Fig. 4) in comparison with other tools. At the same time, SyFi maintains high performance in identifying the presence/absence of genera, species and strains (Table S5) while maintaining a high percentage of pseudoaligned reads (Fig. S7).

## Discussion

As SynCom complexity increases, accurately quantifying bacterial strains using marker gene-based amplicon sequencing becomes more challenging. To address this, we developed SyFi. SyFi is designed to identify intragenomic sequence variation in bacterial genomes for a given marker gene and use the resulting sequence variants to construct genomic fingerprints that in turn can be used to quantify the abundance of microbes in a complex community at heightened taxonomic resolution. Additionally, we show that pseudoalignment of amplicon reads to SyFi-generated fingerprints outperforms classical methods in identifying, distinguishing and quantifying bacterial isolates. Beyond the dissection of complex SynCom datasets, SyFi can be applied to investigate sequence variability in any multi-copy gene.

A computational screen of *16S rRNA* copy number and haplotype distribution across bacterial genomes reveals that genome assemblies often miss small variations in the *16S rRNA* sequence. In this context, SyFi can capture these sequence variations and more accurately determine which *16S rRNA* haplotypes are present compared to retrieving *16S rRNA* sequences directly from genomes using target alignment. The *16S rRNA* copy number distribution generated by SyFi aligns with previous studies [15,16] and indicates for both human gut and plant root microbiomes that bacteria often harbour more than one *16S rRNA* haplotype sequence (Figs2  3). Surprisingly, we discovered few *16S rRNA* haplotypes in complete genomes when retrieving them directly [15], which could indicate that SyFi might be detecting technical errors rather than true biological variation. However, our analysis indicates otherwise. When we ran SyFi on ten closed *Bacillales* genomes – each annotated with only a single *16S rRNA* haplotype – SyFi consistently identified multiple haplotypes across all genomes (Table S4). Sequence alignment of these variants revealed statistically supported nucleotide differences that are consistent with genuine biological variation rather than random sequencing noise (Fig. S5, showing four representative examples). These observations strongly suggest that SyFi is uncovering authentic sequence diversity that is masked or overlooked in the original genome annotations. This highlights a critical limitation in current bacterial genome assemblies, particularly in closed genomes that report only a single *16S rRNA* haplotype. Our findings point to the need for cautious interpretation of *16S rRNA* gene content in public databases and underscore the value of using sensitive tools like SyFi to reassess presumed uniformity in *rRNA* gene sequences. The usage of long reads to concatenate and polish short-read-assembled genomes could potentially lead to the loss of sequence variants across multi-copy marker genes. Despite this, advancements in genome-polishing tools are improving the accuracy of complete bacterial genomes, e.g. by assembling genomes with long reads only instead of short reads and using short reads to infer biological variations subsequently [42]. Such effort makes closed genomes increasingly suitable for complex SynCom studies.

SyFi’s ability to extract accurate *16S rRNA* haplotype profiles from a bacterial genome depends on the quality of the genome assembly and the availability, coverage and accuracy of genomic reads (Appendices 1 and 3). Contaminating sequences in the genome assembly or genomic reads may lead to an overestimation of *16S rRNA* copy numbers or the presence of highly dissimilar intragenomic *16S rRNA* sequences, which might seem unlikely to co-occur in a single genome. Previous studies have shown that the vast majority of *16S rRNA* sequences conform to >95% intra-species sequence identity [46], suggesting that *16S rRNA* haplotypes with <95% sequence identity within a genome are improbable. In rare cases, highly divergent *16S rRNA* sequences may arise due to events such as horizontal gene transfer or the uptake of DNA fragments from the environment [47,48]. Beyond data quality, *16S rRNA* fingerprints may also be subject to copy number overestimation due to the higher G+C content of *16S rRNA* sequences relative to the rest of the genome (Appendix 3). Whether this affects microbiome composition accuracy remains uncertain, as the G+C content-derived biases may be present in both amplicon and metagenomic datasets [49]. Consequently, the G+C content bias in genomic data may be counterbalanced by a similar bias in metagenomic data. Future versions of SyFi may incorporate an optional *16S rRNA* copy number normalization step based on differential G+C content.

Other limitations that SyFi currently harbours are related to the input query sequence and the haplotype calling step [50]. We have not yet assessed whether different *16S rRNA* target sequences significantly alter resulting *16S rRNA* fingerprints. Targeting a shorter or less diverse *16S rRNA* sequence may yield shorter fingerprints and/or lower resolution in distinguishability, potentially influencing microbiome composition as processed through *SyFi quant*. To better understand the impact of target sequence selection on *16S rRNA* fingerprints and microbiome composition, future versions of SyFi could incorporate a phylogeny-dependent marker gene database, allowing target sequence selection based on isolate phylogeny. A set of complete *16S rRNA* sequences spanning the bacterial kingdom will allow the retrieval of the most complete *16S rRNA* sequences for every tested SynCom member. Additionally, SyFi currently employs the ‘WhatsHap phase’ algorithm for *16S rRNA* read phasing, which assumes a diploid model with only two haplotype outcomes. This assumption may lead to an underestimation of the true haplotype diversity within bacterial genomes, especially in cases of extensive biological variation. Despite this limitation, SyFi outperforms benchmark microbiome profiling methods. However, future versions could further improve enhanced accuracy by enabling phasing beyond two haplotypes per sequence, e.g. by using the polyploidy phasing algorithm [51], providing a more precise representation of intragenomic haplotype diversity.

Over the past decade, increasing evidence has highlighted substantial variability in marker genes, such as the *16S rRNA* gene, in terms of copy number and sequence identity. Although rarely accounted for in microbiome profiling, this variability impacts the estimation of bacterial presence and abundance in natural microbiomes. High *16S rRNA* copy numbers can lead to an overestimation of bacterial strain abundance in microbiome datasets [13,15,17, 19], while multiple intragenomic *16S rRNA* variants can result in an overestimation of bacterial species diversity [14]. Therefore, considering marker gene variability in microbiome studies is crucial for improving dataset accuracy. In SynCom studies, the primary objective is to accurately identify and quantify bacteria within the dataset. While SyFi does not perfectly distinguish all bacterial isolates in amplicon sequencing datasets, it represents a significant advancement in microbiome profiling by improving the accuracy of bacterial identification and quantification.

## Supplementary material

10.1099/mgen.0.001461 Uncited Supplementary Material 1.

10.1099/mgen.0.001461 Uncited Supplementary Material 2.

10.1099/mgen.0.001461 Uncited Supplementary Material 3.

10.1099/mgen.0.001461 Uncited Supplementary Material 4.

## Abbreviations

ASV: amplicon sequence variant
MQ: mapping quality
NCBI: National Center for Biotechnology Information
NRMSE: normalized root mean square error
SyFi: SynCom Fingerprinting
SynCom: synthetic community
