# Supplementary material for: SyFi: generating and using sequence fingerprints to distinguish SynCom isolates
Source: Microb Genom. 2025 Sep 4;11(9):001461. doi: 10.1099/mgen.0.001461 (PMC12421257; doi:10.1099/mgen.0.001461)
Supplement: Uncited Supplementary Material 4. [file mgen-11-01461-s004.pdf]

Supplemental information for: “SyFi: generating and using sequence fingerprints to distinguish SynCom isolates”

Selten, Gijs[1]*, Gómez-Repollés, Adrián[2]*, Lamouche, Florian[2,3], Radutoiu, Simona[2], de Jonge, Ronnie[1,4#]

[1] Plant-Microbe Interactions, Department of Biology, Science for Life, Utrecht University, 3584CH, Utrecht, The Netherlands

[2] Department of Molecular Biology and Genetics, Aarhus University, Aarhus, 8000C, Denmark

[3] Present address: University of Angers, Institut Agro, INRAE, IRHS, SFR QUASAV, F-49000 Angers, France

[4] AI Technology for Life, Department of Information and Computing Sciences, Science for Life, Utrecht University, 3584CC, Utrecht, The Netherlands

*These authors contributed equally to the presented work

#Corresponding author: Ronnie de Jonge r.dejonge@uu.nl

Contents

Supplementary data..3
Supplementary figures..4
Supplementary tables..11
Supplementary sequence S1..15
Appendix 1 – *SyFi main* workflow..16
Appendix 2 – SynCom data library preparation..19
Appendix 3 – Contamination, heterogeneity, and GC content affects determination of *16S rRNA* copy number..21
References..23

Supplementary data

Data S1: an accession list of bacterial genomes, WGS read files, and SynCom microbiome samples used in this manuscript.

Data S2: an overview of the 16S rRNA copy number of the plant root bacterial isolates, the shotgun metagenomics microbiome table, the 16S rRNA copy number-normalized microbiome tables of SyFi, Salmon, Qiime2-VSEARCH, Mothur, and Kraken2, and an overview of the proportion of pseudoaligned reads per benchmark method and SyFi.

Data S3: the 16S rRNA copy number-normalized microbiome tables of SyFi, Salmon, Qiime2-VSEARCH, Mothur, and Kraken2 when the references were subsetted to the 382 isolates for which SyFi was able to generate 16S rRNA fingerprints (for Table S5).

Supplementary figures

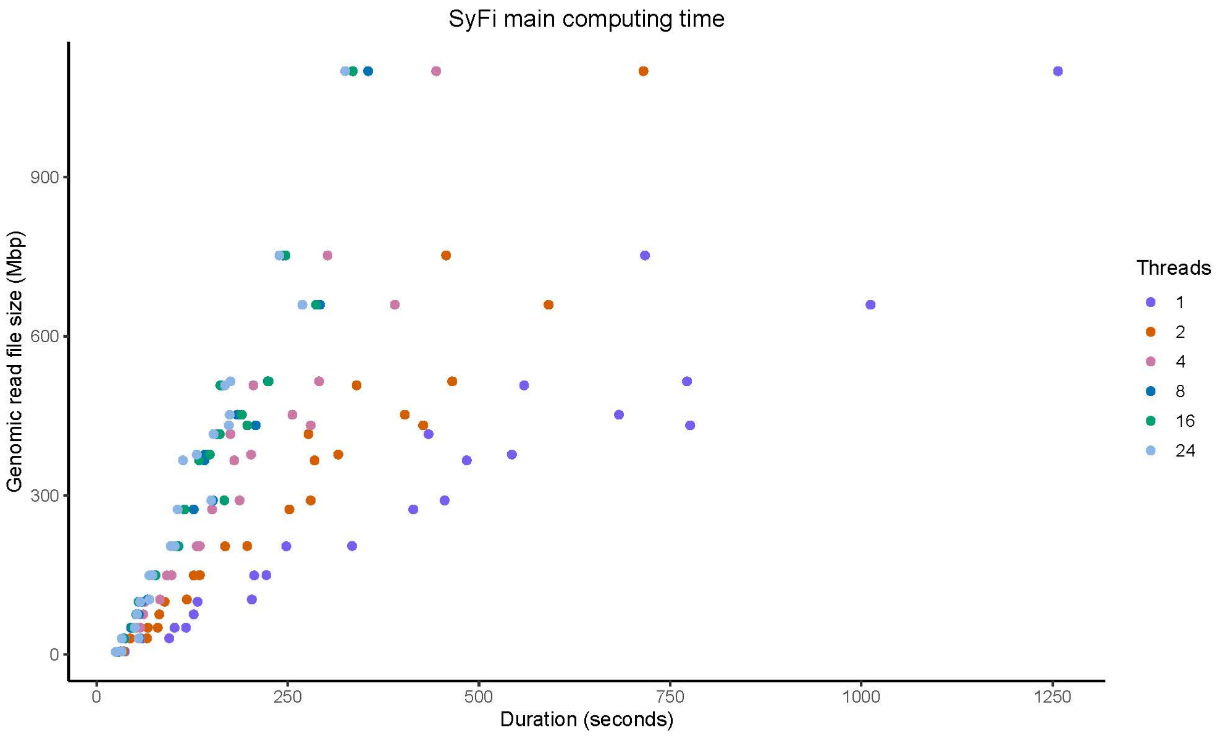

Figure S1. *SyFi main* runtime. The runtime of *SyFi main* (x-axis) is dependent on the size of the genomic read file (y-axis), though can be shortened by using more CPUs.

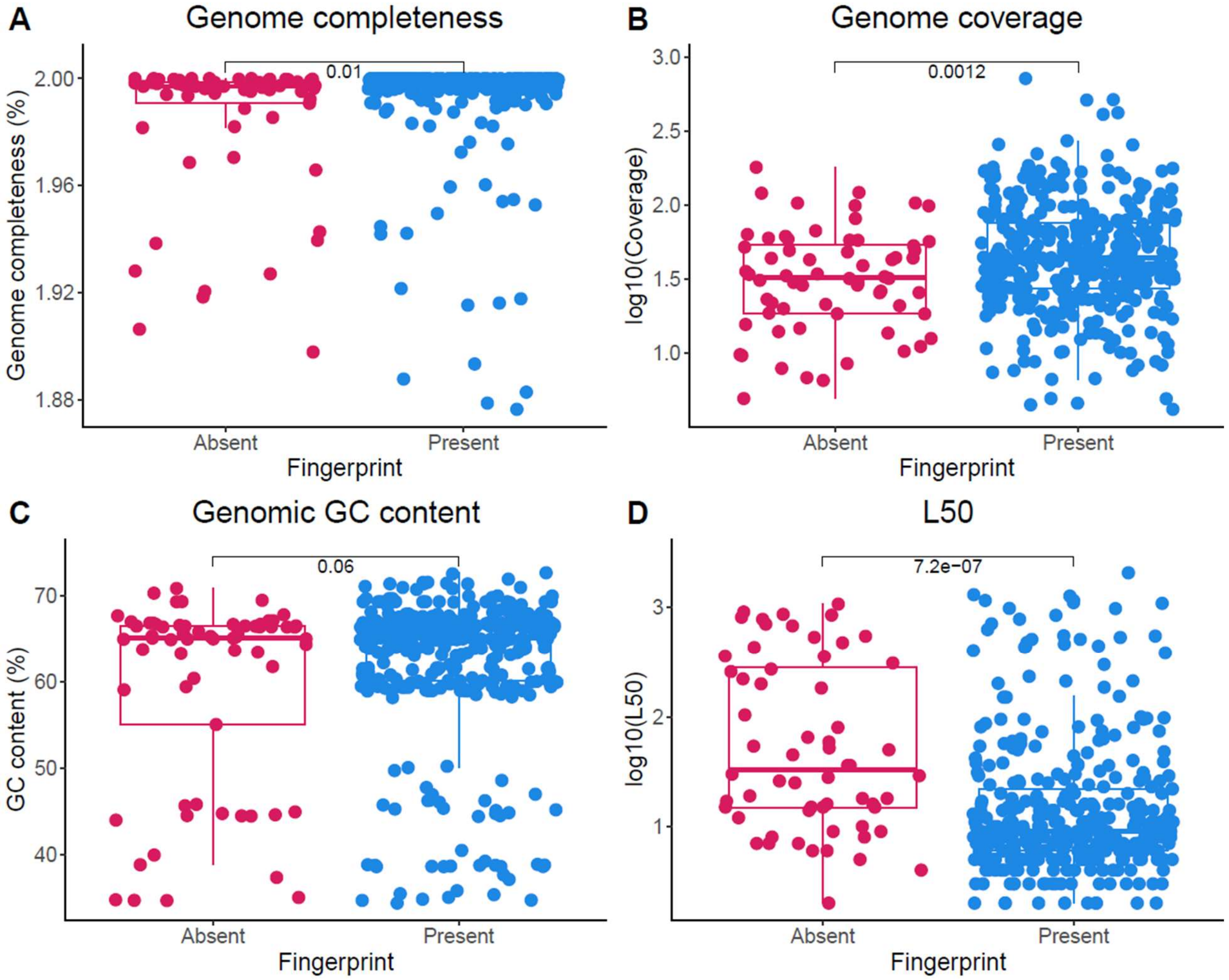

Figure S2. Effect of genome and genome assembly features on SyFi's ability to build a *16S rRNA* fingerprint. Genome completeness (A), coverage (B), GC content (C) and L50 (D) of the genomes for which SyFi was able to build a *16S rRNA* fingerprint (Present) or not (Absent). Statistical differences are tested with a pairwise t-test.

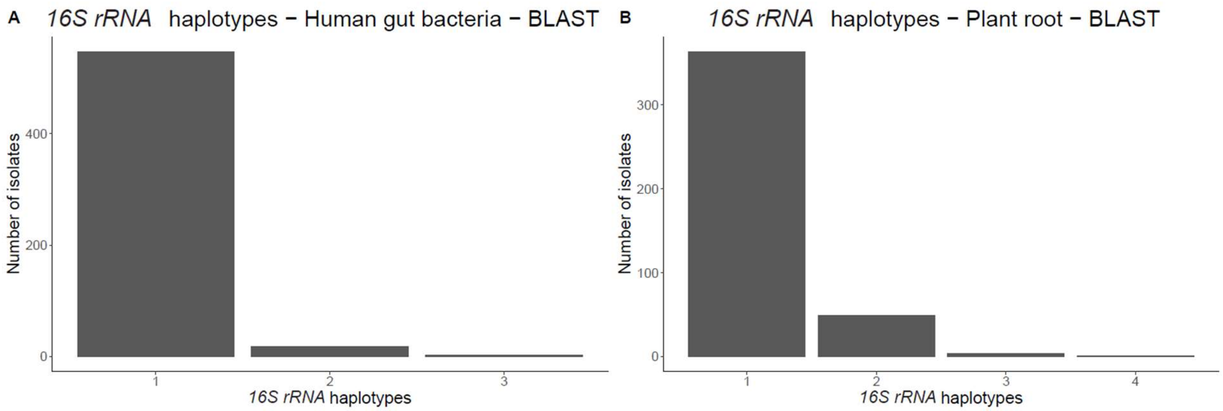

Figure S3. *16S rRNA* haplotype number derived from BLAST. The *16S rRNA* haplotype number from 567 human gut bacterial isolates (A) and 417 plant bacterial genomes (B) when using a direct alignment of the target gene to the bacterial genomes. BLAST alignment hits were filtered using a minimum alignment length threshold of 500 bp to retain both complete and partial variants of the *16S rRNA* gene while excluding short hits derived from small contigs, which may represent technical artifacts such as PCR-related errors.

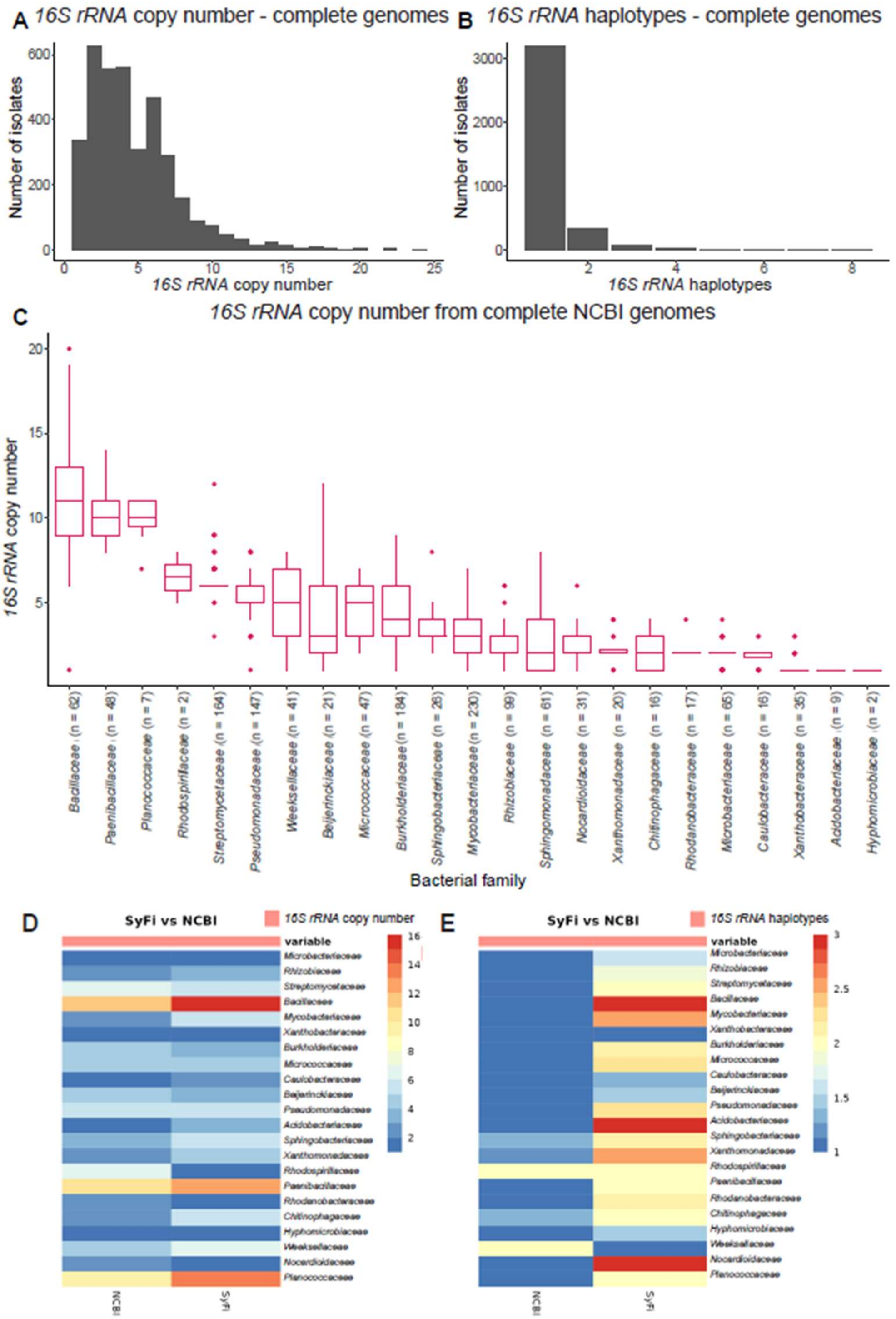

Figure S4. The *16S rRNA* copy number and haplotypes in complete NCBI genomes derived from SyFi. The *16S rRNA* copy number (A) and 1*6S rRNA* haplotypes (B) in 3,615 complete NCBI genomes. The *16S rRNA* copy number per family (C) in the complete NCBI genomes subsetted for families that are present among the 447 isolates (Figure 3C). Comparison of *16S rRNA* copy number (D) and *16S rRNA* haplotypes (E) between the 447 plant root isolates and the 3,615 complete NCBI genomes.

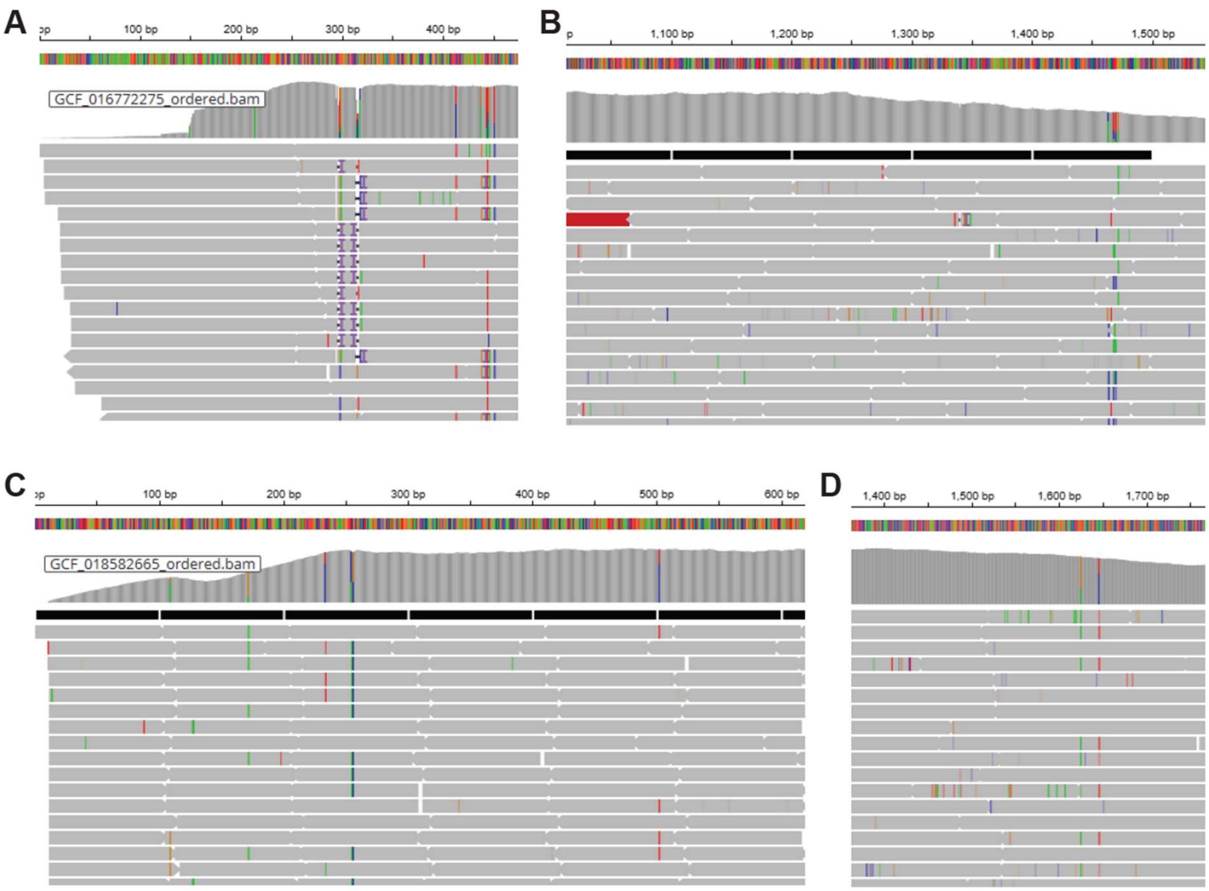

Figure S5. Biological variations in *16S rRNA* sequences of closed Bacillales genomes. Despite indicating one *16S rRNA* haplotype in the genome assembly, SyFi rightly finds multiple haplotypes clearly indicated by the presence of biological variations, either at the beginning of the sequence (A and C – GCF_016777275 and GCF_018582665) or at the end (B and D – GCF_030123445 and GCF_004124315). All panels are snapshots from IGV.

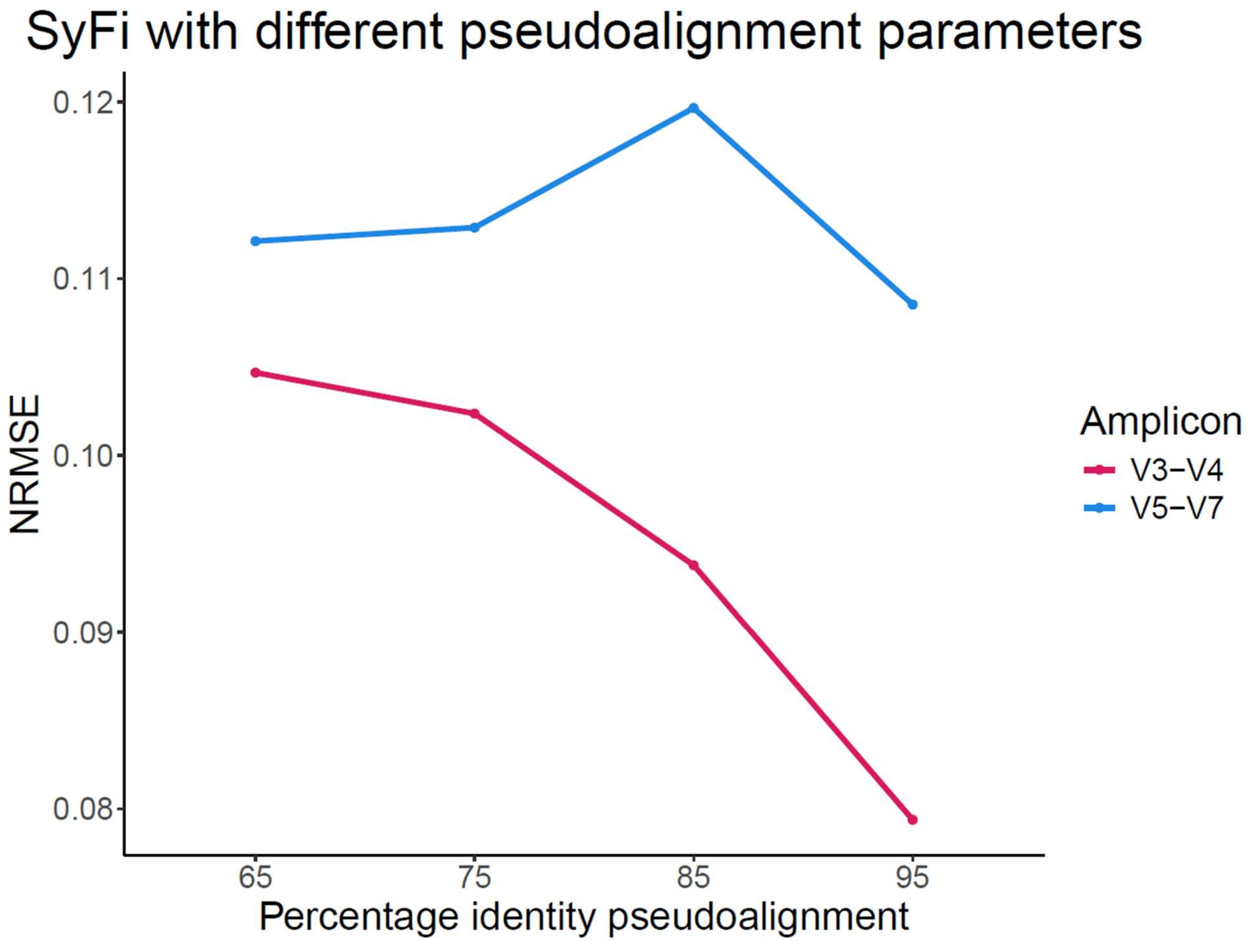

Figure S6. SyFi's weighted accuracy with different sequence percentage identity in pseudoalignment of metagenomic reads to *16S rRNA* V3-V4 or V5-V7 fingerprints. SyFi's weighted accuracy is assessed by computing the error to the shotgun metagenome dataset (NRMSE value) (y-axis) for each pseudoalignment percentage identity that is used in Salmon to match metagenomic reads with *16S rRNA* V3-V4 or V5-V7 fingerprints (x-axis).

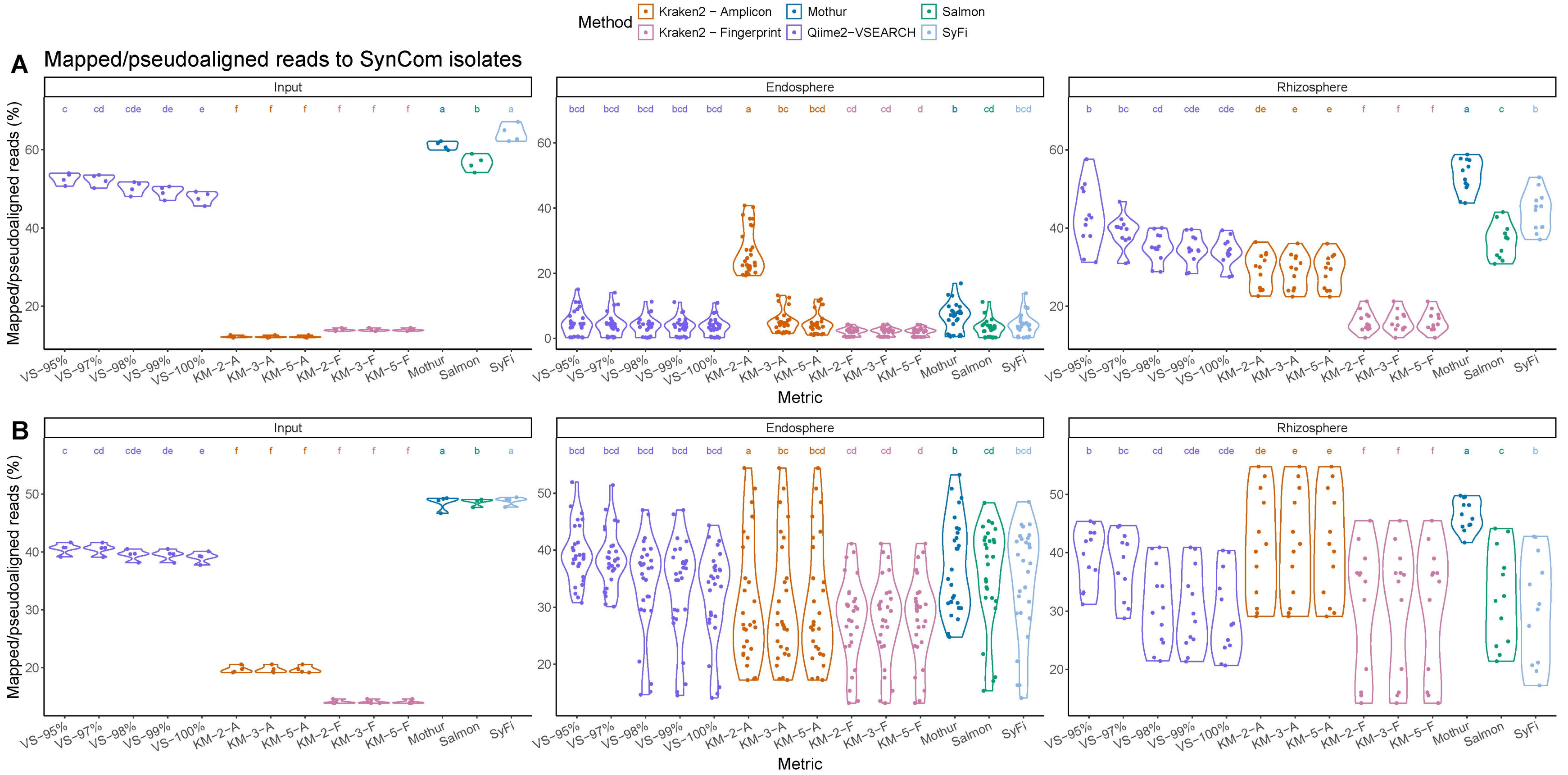

Figure S7. Percentages of mapped or pseudoaligned reads in the V3-V4 (A) and V5-V7 (B) datasets for microbial culture (Input), endosphere and rhizosphere samples. The relative amount of reads that are mapped to amplicon or fingerprints sequences is shown for Qiime2-VSEARCH, Kraken2, Mothur, direct pseudoalignment of amplicon reads using Salmon, and SyFi. For Qiime2-VSEARCH and Kraken2 different thresholds of mapping are implemented, either by sequence identity (Qiime2-VSEARCH) or minimum number of exact K-mer matches (Kraken2). Statistical differences are indicated by ANOVAs and post-hoc tests ($p < 0.05$).

Supplementary tables

Table S1. Sample overview for the SyFi validation, derived from Selten *et al.* (2024a).

Sample ID	Host	Compartment	Nutrient condition	Replicate
**Input_AtSC_B1**	-	Input	-	B1
**Input_AtSC_B2**	-	Input	-	B2
**Input_AtSC_B3**	-	Input	-	B3
**Input_AtSC_B4**	-	Input	-	B4
**Lj_AtSC_RZ_high_B1**	*Lotus japonicus*	Rhizosphere	Long Ashton medium - high NPK	B1
**Lj_AtSC_RZ_high_B2**	*Lotus japonicus*	Rhizosphere	Long Ashton medium - high NPK	B2
**Lj_AtSC_RZ_high_B3**	*Lotus japonicus*	Rhizosphere	Long Ashton medium - high NPK	B3
**Lj_AtSC_RZ_high_B4**	*Lotus japonicus*	Rhizosphere	Long Ashton medium - high NPK	B4
**Lj_AtSC_ES_high_B1**	*Lotus japonicus*	Endosphere	Long Ashton medium - high NPK	B1
**Lj_AtSC_ES_high_B2**	*Lotus japonicus*	Endosphere	Long Ashton medium - high NPK	B2
**Lj_AtSC_ES_high_B3**	*Lotus japonicus*	Endosphere	Long Ashton medium - high NPK	B3
**Lj_AtSC_ES_high_B4**	*Lotus japonicus*	Endosphere	Long Ashton medium - high NPK	B4
**At_AtSC_ES_high_B1**	*Arabidopsis thaliana*	Endosphere	Long Ashton medium - high NPK	B1
**At_AtSC_ES_high_B2**	*Arabidopsis thaliana*	Endosphere	Long Ashton medium - high NPK	B2
**At_AtSC_ES_high_B3**	*Arabidopsis thaliana*	Endosphere	Long Ashton medium - high NPK	B3
**At_AtSC_ES_high_B4**	*Arabidopsis thaliana*	Endosphere	Long Ashton medium - high NPK	B4
**Hv_AtSC_RZ_high_B1**	*Hordeum vulgare*	Rhizosphere	Long Ashton medium - high NPK	B1
**Hv_AtSC_RZ_high_B2**	*Hordeum vulgare*	Rhizosphere	Long Ashton medium - high NPK	B2
**Hv_AtSC_RZ_high_B3**	*Hordeum vulgare*	Rhizosphere	Long Ashton medium - high NPK	B3
**Hv_AtSC_RZ_high_B4**	*Hordeum vulgare*	Rhizosphere	Long Ashton medium - high NPK	B4
**Hv_AtSC_ES_high_B1**	*Hordeum vulgare*	Endosphere	Long Ashton medium - high NPK	B1
**Hv_AtSC_ES_high_B2**	*Hordeum vulgare*	Endosphere	Long Ashton medium - high NPK	B2
**Hv_AtSC_ES_high_B3**	*Hordeum vulgare*	Endosphere	Long Ashton medium - high NPK	B3
**Hv_AtSC_ES_high_B4**	*Hordeum vulgare*	Endosphere	Long Ashton medium - high NPK	B4
**Lj_AtSC_RZ_low_B1**	*Lotus japonicus*	Rhizosphere	Long Ashton medium - low NPK	B1
**Lj_AtSC_RZ_low_B2**	*Lotus japonicus*	Rhizosphere	Long Ashton medium - low NPK	B2
**Lj_AtSC_RZ_low_B3**	*Lotus japonicus*	Rhizosphere	Long Ashton medium - low NPK	B3
**Lj_AtSC_RZ_low_B4**	*Lotus japonicus*	Rhizosphere	Long Ashton medium - low NPK	B4
**Lj_AtSC_ES_low_B1**	*Lotus japonicus*	Endosphere	Long Ashton medium - low NPK	B1
**Lj_AtSC_ES_low_B2**	*Lotus japonicus*	Endosphere	Long Ashton medium - low NPK	B2
**Lj_AtSC_ES_low_B3**	*Lotus japonicus*	Endosphere	Long Ashton medium - low NPK	B3
**Lj_AtSC_ES_low_B4**	*Lotus japonicus*	Endosphere	Long Ashton medium - low NPK	B4
**At_AtSC_ES_low_B1**	*Arabidopsis thaliana*	Endosphere	Long Ashton medium - low NPK	B1
**At_AtSC_ES_low_B2**	*Arabidopsis thaliana*	Endosphere	Long Ashton medium - low NPK	B2
**At_AtSC_ES_low_B3**	*Arabidopsis thaliana*	Endosphere	Long Ashton medium - low NPK	B3
**At_AtSC_ES_low_B4**	*Arabidopsis thaliana*	Endosphere	Long Ashton medium - low NPK	B4
**Hv_AtSC_ES_low_B1**	*Hordeum vulgare*	Endosphere	Long Ashton medium - low NPK	B1
**Hv_AtSC_ES_low_B2**	*Hordeum vulgare*	Endosphere	Long Ashton medium - low NPK	B2
**Hv_AtSC_ES_low_B3**	*Hordeum vulgare*	Endosphere	Long Ashton medium - low NPK	B3
**Hv_AtSC_ES_low_B4**	*Hordeum vulgare*	Endosphere	Long Ashton medium - low NPK	B4
**At_AtSC_ES_HL_orig_B1**	*Arabidopsis thaliana*	Endosphere	Hoagland medium	B1
**At_AtSC_ES_HL_orig_B2**	*Arabidopsis thaliana*	Endosphere	Hoagland medium	B2
**At_AtSC_ES_HL_orig_B3**	*Arabidopsis thaliana*	Endosphere	Hoagland medium	B3
**At_AtSC_ES_HL_orig_B4**	*Arabidopsis thaliana*	Endosphere	Hoagland medium	B4

Table S2. Explanation of pseudoalignment for distinguishing highly similar sequences. When pseudoaligning genomic reads from a computationally modified *Pseudomonas protegens* CHA0 to the wildtype strain and the modified strain, we find 98.7% of the reads to be correctly pseudoaligned.

Target ID	Fingerprint overview	Length	Effective Length	Estimated counts	%
***Pseudomonas protegens* CHA0**	H1-H1-H1-H1-H1	7,730	7,731	55.0971	1.28543
**Modified *Pseudomonas protegens* CHA0**	H1-H1-H1-H1-H2	7,724	7,725	4,227.9	98.7146

Table S3. VSEARCH Clustering of V3-V4 and V5-V7 amplicon sequences and SyFi fingerprints on the plant root bacterial isolate dataset. The rows indicate whether VSEARCH was run on the original sequences that were retrieved directly from the genome clustered at different percentage identities (95, 97, 98, 99, 100% sequence identity) or on the SyFi fingerprints clustered at 100% sequence identity.

VSEARCH clustering percentage (%)	Metric	No. of clusters	No. of singletons	No. of isolates in largest cluster	Average no. of isolates in cluster	No. of isolates
**95**	V3-V4	38	10	60	10.2	**387**
**97**	V3-V4	57	19	50	6.8	**387**
**98**	V3-V4	63	22	40	6.1	**387**
**99**	V3-V4	84	36	37	4.6	**387**
**100**	V3-V4	125	69	28	3.1	**387**
**SyFi**	V3-V4	**161**	**102**	**24**	**2.4**	382
**95**	V5-V7	47	17	63	8.3	390
**97**	V5-V7	60	19	63	6.5	390
**98**	V5-V7	73	32	63	5.3	390
**99**	V5-V7	92	46	44	4.2	390
**100**	V5-V7	126	72	27	3.1	390
**SyFi**	V5-V7	**157**	**100**	**27**	**2.4**	**382**

Table S4. *16S rRNA* copy number and haplotype number in ten Bacillales NCBI complete genomes. These numbers are either found by direct target alignment of the *16S rRNA* sequence of *Paenisporosarcina* (Sequence S1) to the genomes or by running SyFi on these genomes. Evidently SyFi finds more *16S rRNA* haplotypes than BLAST, also illustrated by the number of variants found among the *16S rRNA* copies.

NCBI Bacillales strain	BLAST - *16S rRNA* copy number	BLAST - *16S* rRNA haplotype	SyFi – *16S rRNA* copy number	SyFi - *16S* rRNA haplotype	No of variants
**GCF_030292135**	14	1	11	2	18
**GCF_004124315**	10	1	10	2	17
**GCF_030167035**	14	1	8	2	4
**GCF_018582665**	10	1	8	2	9
**GCF_017809215**	12	2	15	2	16
**GCF_016772275**	10	1	10	2	33
**GCF_030123445**	13	1	8	2	37
**GCF_032818155**	10	1	14	2	5
**GCF_016724865**	14	1	10	2	12
**GCF_030123485**	14	1	8	2	8

Table S5. SyFi's accuracy in assessment of presence/absence of isolates. The percentage of genera (top), species (middle) and strains (bottom) that SyFi correctly identified as present (TP), absent (TN), as well as incorrectly identified as present (FP) or absent (FN) in comparison with other tools. Other tools include Qiime2-VSEARCH (with sequences clustered at 95, 97, 98, 99, or 100% sequence identity), Mothur, and Kraken2 (with two, three, or five minimum hit groups to identify isolates) run on both the BLAST-retrieved ASV sequences (Amplicon) and the SyFi-generated fingerprints. The accuracy, precision, recall, and taxon specificity is indicated in the last column with bold values with the highest performance.

Genus	TP	FP	TN	FN	Accuracy	Precision	Recall/Taxon sensitivity	Taxon Specificity
**VS_95%_V3V4**	20.70%	1.60%	69%	8.70%	89.70%	92.83%	70.41%	97.73%
**VS_97%_V3V4**	21.30%	1.50%	69.10%	8%	90.49%	93.42%	72.70%	97.88%
**VS_98%_V3V4**	21.50%	0.50%	70.10%	7.80%	91.69%	**97.73%**	73.38%	**99.29%**
**VS_99%_V3V4**	21.20%	0.60%	70.10%	8.10%	91.30%	97.25%	72.35%	99.15%
**VS_100%_V3V4**	19.50%	0.60%	70%	9.90%	89.50%	97.01%	66.33%	99.15%
**Salmon_V3V4**	23.10%	3.20%	67.40%	6.20%	90.59%	87.83%	78.84%	95.47%
**SyFi_V3V4**	23.60%	0.90%	69.80%	5.80%	**93.31%**	96.33%	**80.27%**	98.73%
**Mothur_V3V4**	22.80%	1.70%	69%	6.60%	91.71%	93.06%	77.55%	97.60%
**Kraken2:KM-2-A_V3V4**	12.80%	16.40%	54.20%	16.60%	67.00%	43.84%	43.54%	76.77%
**Kraken2:KM-3-A_V3V4**	12.80%	16.60%	54%	16.60%	66.80%	43.54%	43.54%	76.49%
**Kraken2:KM-5-A_V3V4**	12.80%	16.90%	53.70%	16.60%	66.50%	43.10%	43.54%	76.06%
**Kraken2:KM-2-F_V3V4**	14.60%	16.60%	54%	14.80%	68.60%	46.79%	49.66%	76.49%
**Kraken2:KM-3-F_V3V4**	14.60%	16.60%	54%	14.80%	68.60%	46.79%	49.66%	76.49%
**Kraken2:KM-5-F_V3V4**	14.80%	17%	53.70%	14.60%	68.43%	46.54%	50.34%	75.95%
**VS_95%_V5V7**	21.10%	2.40%	68.30%	8.30%	89.31%	89.79%	71.77%	96.61%
**VS_97%_V5V7**	20.90%	2.20%	68.40%	8.50%	89.30%	90.48%	71.09%	96.88%
**VS_98%_V5V7**	22.80%	1.20%	69.40%	6.60%	92.20%	95.00%	**77.55%**	98.30%
**VS_99%_V5V7**	22.70%	0.70%	69.90%	6.70%	**92.60%**	**97.01%**	77.21%	**99.01%**
**VS_100%_V5V7**	22.80%	0.80%	69.80%	6.60%	**92.60%**	96.61%	**77.55%**	98.87%
**Salmon_V5V7**	18.80%	3.60%	67%	10.60%	85.80%	83.93%	63.95%	94.90%
**SyFi_V5V7**	22.60%	1.50%	69.10%	6.70%	91.79%	93.78%	77.13%	97.88%
**Mothur_V5V7**	23.20%	2.60%	68%	6.20%	91.20%	89.92%	78.91%	96.32%
**Kraken2:KM-2-A_V5V7**	16.60%	14.80%	55.80%	12.80%	72.40%	52.87%	56.46%	79.04%
**Kraken2:KM-3-A_V5V7**	16.60%	14.80%	55.80%	12.80%	72.40%	52.87%	56.46%	79.04%
**Kraken2:KM-5-A_V5V7**	16.60%	14.80%	55.80%	12.80%	72.40%	52.87%	56.46%	79.04%
**Kraken2:KM-2-F_V5V7**	12.60%	16.40%	54.20%	16.70%	66.87%	43.45%	43.00%	76.77%
**Kraken2:KM-3-F_V5V7**	12.60%	16.40%	54.20%	16.70%	66.87%	43.45%	43.00%	76.77%
**Kraken2:KM-5-F_V5V7**	12.60%	16.40%	54.20%	16.70%	66.87%	43.45%	43.00%	76.77%

Species	TP	FP	TN	FN	Accuracy	Precision	Recall/Taxon sensitivity	Taxon Specificity
**VS_95%_V3V4**	7.90%	1.90%	83.90%	6.30%	91.80%	80.61%	55.63%	97.79%
**VS_97%_V3V4**	8.30%	1.80%	84%	5.80%	92.39%	82.18%	58.87%	97.90%
**VS_98%_V3V4**	8.40%	1.50%	84.30%	5.80%	92.70%	84.85%	59.15%	98.25%
**VS_99%_V3V4**	8.20%	1.40%	84.40%	5.90%	92.69%	85.42%	58.16%	98.37%
**VS_100%_V3V4**	7.50%	1.20%	84.60%	6.60%	92.19%	**86.21%**	53.19%	**98.60%**
**Salmon_V3V4**	9.30%	3.20%	82.60%	4.90%	91.90%	74.40%	65.49%	96.27%
**SyFi_V3V4**	9.90%	2%	83.80%	4.30%	**93.70%**	83.19%	**69.72%**	97.67%
**Mothur_V3V4**	8.50%	3.10%	82.80%	5.60%	91.30%	73.28%	60.28%	96.39%
**Kraken2:KM-2-A_V3V4**	3.30%	8.90%	76.90%	10.90%	80.20%	27.05%	23.24%	89.63%
**Kraken2:KM-3-A_V3V4**	3.30%	9%	76.80%	10.90%	80.10%	26.83%	23.24%	89.51%
**Kraken2:KM-5-A_V3V4**	3.30%	9.10%	76.70%	10.90%	80.00%	26.61%	23.24%	89.39%
**Kraken2:KM-2-F_V3V4**	3.70%	9.30%	76.60%	10.50%	80.22%	28.46%	26.06%	89.17%
**Kraken2:KM-3-F_V3V4**	3.70%	9.30%	76.60%	10.50%	80.22%	28.46%	26.06%	89.17%
**Kraken2:KM-5-F_V3V4**	3.70%	9.50%	76.30%	10.50%	80.00%	28.03%	26.06%	88.93%
**VS_95%_V5V7**	8.10%	1.90%	83.90%	6.10%	92.00%	81.00%	57.04%	97.79%
**VS_97%_V5V7**	8.10%	1.80%	84%	6%	92.19%	81.82%	57.45%	97.90%
**VS_98%_V5V7**	9%	1.60%	84.20%	5.20%	93.20%	84.91%	**63.38%**	98.14%

VS_99%_V5V7	9%	1.50%	84.40%	5.20%	**93.31%**	**85.71%**	**63.38%**	**98.25%**
VS_100%_V5V7	9%	1.50%	84.30%	5.20%	93.30%	**85.71%**	**63.38%**	**98.25%**
Salmon_V5V7	7.10%	3.30%	82.50%	7%	89.69%	68.27%	50.35%	96.15%
SyFi_V5V7	8.90%	1.80%	84%	5.30%	92.90%	83.18%	62.68%	97.90%
Mothur_V5V7	7.50%	1.80%	84%	6.60%	91.59%	80.65%	53.19%	97.90%
Kraken2:KM-2-A_V5V7	3.10%	8.40%	77.40%	11.10%	80.50%	26.96%	21.83%	90.21%
Kraken2:KM-3-A_V5V7	3.10%	8.40%	77.40%	11.10%	80.50%	26.96%	21.83%	90.21%
Kraken2:KM-5-A_V5V7	3.10%	8.40%	77.40%	11.10%	80.50%	26.96%	21.83%	90.21%
Kraken2:KM-2-F_V5V7	2.30%	8%	77.80%	11.90%	80.10%	22.33%	16.20%	90.68%
Kraken2:KM-3-F_V5V7	2.30%	8%	77.80%	11.90%	80.10%	22.33%	16.20%	90.68%
Kraken2:KM-5-F_V5V7	2.30%	8%	77.80%	11.90%	80.10%	22.33%	16.20%	90.68%

Strain	TP	FP	TN	FN	Accuracy	Precision	Recall/Taxon sensitivity	Taxon Specificity
VS_95%_V3V4	22.80%	2%	63.90%	11.30%	86.70%	91.94%	66.86%	96.97%
VS_97%_V3V4	18.80%	2%	69.80%	9.40%	88.60%	90.38%	66.67%	97.21%
VS_98%_V3V4	18.80%	1.10%	72%	8.10%	90.80%	**94.47%**	69.89%	98.50%
VS_99%_V3V4	14.40%	1%	76.70%	8%	91.01%	93.51%	64.29%	**98.71%**
VS_100%_V3V4	9.70%	1.10%	81.10%	8.10%	90.80%	89.81%	54.49%	98.66%
Salmon_V3V4	12.50%	2.40%	79.80%	5.30%	92.30%	83.89%	**70.22%**	97.08%
SyFi_V3V4	10.30%	2.10%	82.50%	5.10%	**92.80%**	83.06%	66.88%	97.52%
Mothur_V3V4	11.20%	2.60%	79.60%	6.60%	90.80%	81.16%	62.92%	96.84%
Kraken2:KM-2-A_V3V4	3.40%	11.50%	70.70%	14.50%	74.03%	22.82%	18.99%	86.01%
Kraken2:KM-3-A_V3V4	3.40%	11.60%	70.60%	14.50%	73.93%	22.67%	18.99%	85.89%
Kraken2:KM-5-A_V3V4	3.40%	11.70%	70.50%	14.50%	73.83%	22.52%	18.99%	85.77%
Kraken2:KM-2-F_V3V4	3.60%	10.60%	74%	11.70%	77.68%	25.35%	23.53%	87.47%
Kraken2:KM-3-F_V3V4	3.60%	10.60%	74%	11.70%	77.68%	25.35%	23.53%	87.47%
Kraken2:KM-5-F_V3V4	3.60%	10.90%	73.80%	11.70%	77.40%	24.83%	23.53%	87.13%
VS_95%_V5V7	20.90%	2.50%	68%	8.60%	88.90%	89.32%	70.85%	96.45%
VS_97%_V5V7	16.70%	1.90%	74.20%	7.30%	90.81%	89.78%	69.58%	97.50%
VS_98%_V5V7	17%	0.70%	77.20%	5.10%	94.20%	**96.05%**	**76.92%**	**99.10%**
VS_99%_V5V7	14%	1%	79.80%	5.20%	93.80%	93.33%	72.92%	98.76%
VS_100%_V5V7	11.30%	1.40%	83.10%	4.20%	**94.40%**	88.98%	72.90%	98.34%
Salmon_V5V7	10.60%	3.40%	81.20%	4.90%	91.71%	75.71%	68.39%	95.98%
SyFi_V5V7	8.90%	2.20%	83.10%	5.70%	92.09%	80.18%	60.96%	97.42%
Mothur_V5V7	9.30%	1.40%	83%	6.30%	92.30%	86.92%	59.62%	98.34%
Kraken2:KM-2-A_V5V7	1.90%	11.60%	72.90%	13.50%	74.87%	14.07%	12.34%	86.27%
Kraken2:KM-3-A_V5V7	1.90%	11.60%	72.90%	13.50%	74.87%	14.07%	12.34%	86.27%
Kraken2:KM-5-A_V5V7	1.90%	11.60%	72.90%	13.50%	74.87%	14.07%	12.34%	86.27%
Kraken2:KM-2-F_V5V7	2.50%	8.30%	77.10%	12.10%	79.60%	23.15%	17.12%	90.28%
Kraken2:KM-3-F_V5V7	2.50%	8.30%	77.10%	12.10%	79.60%	23.15%	17.12%	90.28%
Kraken2:KM-5-F_V5V7	2.50%	8.30%	77.10%	12.10%	79.60%	23.15%	17.12%	90.28%

Supplementary sequence S1

>*Paenisporosarcina* sp. *16S rRNA*
AGAGTTTGATCCTGGCTCAGGACGAACGCTGGCGGCGTGCCTAATACATGCAAGTCGAGCGGAATGATGAAGAA
GCTTGCTTCTTCTGATTTTAGCGGCGGACGGGTGAGTAACACGTGGGCAACCTACCTTGTAGATTGGGATAACT
CCGGGAAACCGGGGCTAATACCGAATAATCCATTTTGCTTCATGGCAAGATGTTGAAAGGCGGCTTCGGCTGTC
ACTACGAGATGGGCCCGCGGCGTATTAGCTAGTTGGTAGGGTAATGGCCTACCAAGGCGACGATACGTAGCCGA
CCTGAGAGGGTGATCGGCCACACTGGGACTGAGACACGGCCCAGACTCCTACGGGAGGCAGCAGTAGGGAATCT
TCCACAATGGACGAAAGTCTGATGGAGCAACGCCGCGTGAGTGAAGAAGGTTTTCGGATCGTAAAACTCTGTTG
TAAGGGAAGAACACGTACGAGAGTAACTGCTCGTACCTTGACGGTACCTTATTAGAAAGCCACGGCTAACTACG
TGCCAGCAGCCGCGGTAATACGTAGGTGGCAAGCGTTGTCCGGAATTATTGGGCGTAAAGCGCGCGCAGGCGGT
TCTTTAAGTCTGATGTGAAAGCCCACGGCTCAACCGTGGAGGGTCATTGGAAACTGGAGAACTTGAGTACAGAA
GAGGAAAGCGGAATTCCACGTGTAGCGGTGAAATGCGTAGAGATGTGGAGGAACACCAGTGGCGAAGGCGGCTT
TCTGGTCTGTAACTGACGCTGAGGCGCGAAAGCGTGGGGAGCAAACAGGATTAGATACCCTGGTAGTCCACGCC
GTAAACGATGAGTGCTAAGTGTTAGGGGGTTTCCGCCCCTTAGTGCTGCAGCTAACGCATTAAGCACTCCGCCT
GGGGAGTACGACCGCAAGGTTGAAACTCAAAGGAATTGACGGGGGCCCGCACAAGCGGTGGAGCATGTGGTTTA
ATTCGAAGCAACGCGAAGAACCTTACCAGGTCTTGACATCCCACTGACCGGCTTAGAGATAGGCTTTTCCCTTC
GGGGACAGTGGTGACAGGTGGTGCATGGTTGTCGTCAGCTCGTGTCGTGAGATGTTGGGTTAAGTCCCGCAACG
AGCGCAACCCTTGATCTTAGTTGCCAGCATTCAGTTGGGCACTCTAAGGTGACTGCCGGTGACAAACCGGAGGA
AGGTGGGGATGACGTCAAATCATCATGCCCCTTATGACCTGGGCTACACACGTGCTACAATGGACGATACAAAG
GGCTGCAAACCCGCGAGGGGGAGCCAATCCCATAAAATCGTTCTCAGTTCGGATTGTAGGCTGCAACTCGCCTA
CATGAAGCCGGAATCGCTAGTAATCGTGGATCAGCATGCCACGGTGAATACGTTCCCGGGCCTTGTACACACCG
CCCGTCACACCACGAGAGTTTGTAACACCCGAAGTCGGTGAGGTAACCTTTACAGGAGCCAGCCGCCGAAGGTG
GGACAGATGATTGGGGTGAAGTCGTAACAAGGTAGCCGTATCGGAAGGTGCGGCTGGATCACCT

Appendix 1 – *SyFi main* workflow

In *SyFi main*, a fingerprint of the target sequence for every bacterial genome is built (Figure 1). For this SyFi requires the genome sequence (fasta format), genomic single or paired end reads (fastq format), and a target sequence (fasta format) as input. *SyFi main*'s construction of the fingerprint can be divided into three steps.

In the first step, SyFi aligns the target sequence with the bacterial genome in a nucleotide-nucleotide BLAST alignment (v2.13.0) (default parameters) (Altschul et al., 1990) and extracts the nucleotide sequence with the highest identity score. Subsequently, the genomic reads are mapped to this sequence using BWA with default parameters (v2.2.1) (Li & Durbin, 2009) and filtered to obtain all the corresponding read pairs using Samtools (v1.16.1) (Li et al., 2009). The target sequence is then reassembled using SPAdes with default parameters (v3.15.5) (Bankevich et al., 2012) as SPAdes does not allow ambiguous bases in the target assembly. Biological variations, like SNPs, are masked by ambiguous nucleotides and should therefore be avoided. These SPAdes-assembled sequences are subsequently subjected to a length threshold to filter out sequences that are too small, after which the target sequence reads are filtered and trimmed according to the target sequence length by Samtools (v1.16.1) (Li et al., 2009). These steps are added to gain a clean target sequence from the genome with clean corresponding genomic reads.

In the second step, SyFi uses the target sequence and corresponding reads to call biological variations in the target sequence using the Picard algorithm (v2.27.5) in the GATK software (v3.8) (McKenna et al., 2010). After calling the variants in the target sequence, SyFi will enter three different modes depending on whether biological variations were found in the target sequence. When the variant calling workflow finds variants in the target sequence, the workflow will enter 'Mode 1' (Figure 1). If no variants are found, but SPAdes was already able to build multiple haplotypes, the target sequences will directly be forwarded to the Kallisto step in 'Mode 2'. When there is only one target sequence with no variants after the variant calling workflow, it indicates the bacterial isolate has only one haplotype. SyFi will then proceed in 'Mode 3' and calculate the copy number of this one haplotype before creating the fingerprint.

In Mode 1, Whatshap is used to identify biological variations that co-occur in the target sequence, which may indicate different variants, or haplotypes, of the target sequence (v1.7) (Martin et al., 2016). If there remain any identical haplotypes or haplotypes that are smaller than the length threshold, these are removed after the Whatshap step.

The third and last step of SyFi is to construct the fingerprint from the discovered haplotypes. In the case of Mode 1 and Mode 2, the transcript quantifying tool Kallisto (v0.48.0) (Bray et al., 2016) (default parameters) is employed to calculate the coverage of each target gene haplotype within the genome. This is calculated by pseudoalignment of genomic reads to the different haplotype sequences. In the meantime, the target gene copy number is estimated by comparing the target gene coverage to the genomic coverage. Together with the individual haplotype coverage values, these are used to estimate the occurrence of each haplotype in the bacterial genome. In the case of Mode 3, the target gene copy number indicates directly the occurrence of the only found haplotype.

In this copy number estimation, SyFi implements a threshold for the maximum number of copies. This can lead to the removal of haplotypes that occur in extremely low frequencies, which are likely biological contaminations or technical artifacts, either stemming from Whatshap (biological variations that might not occur together) or from sequencing errors. Table 1 shows an example on how the copy

number of target sequences and haplotypes are estimated. Finally, the creation of a unique fingerprint is achieved by concatenating all the haplotypes into a fasta file according to their occurrences.

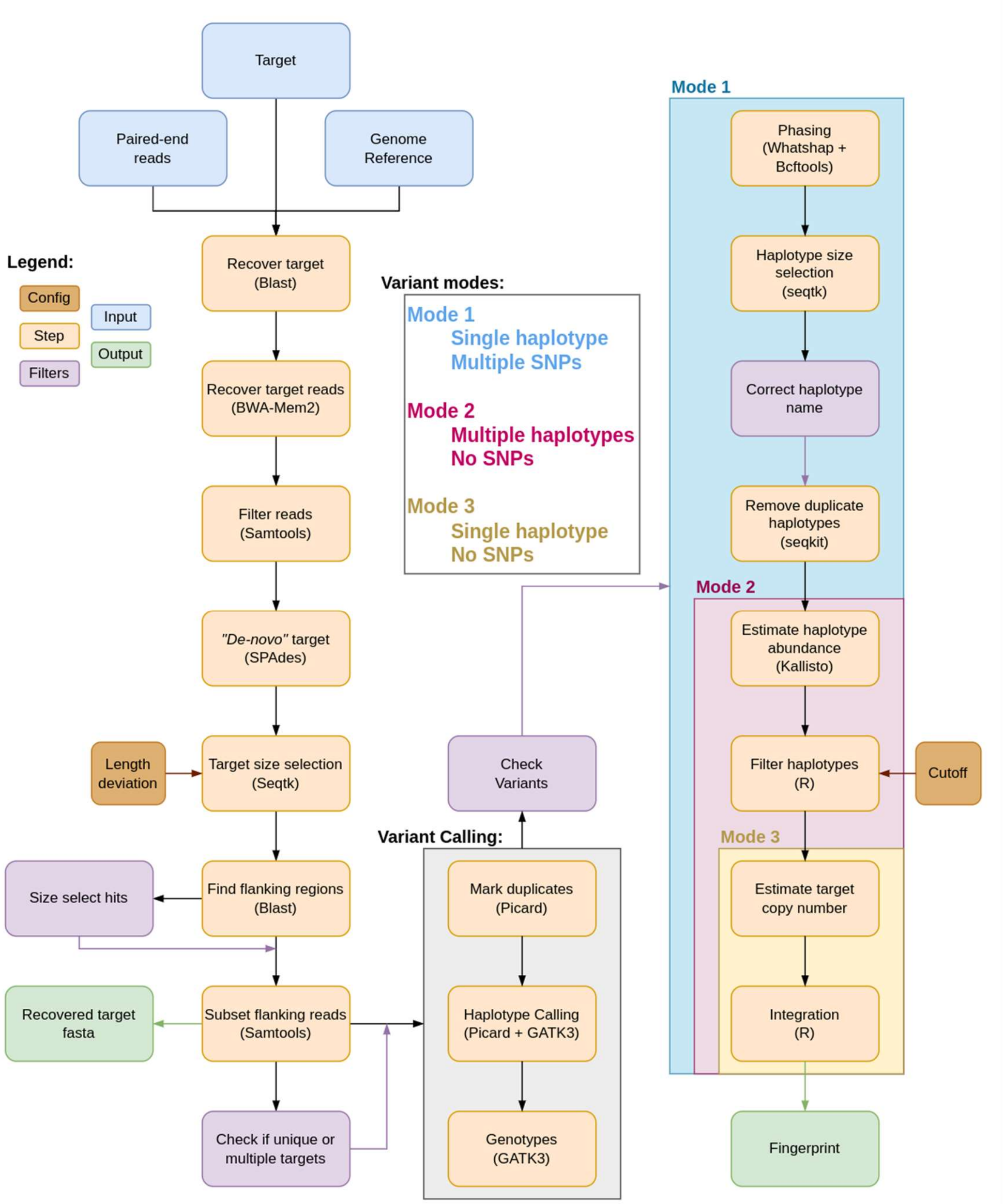

Figure 1. **Detailed overview of *SyFi main*. See text under Appendix 1 for clear explanation of all the steps.**

Table 1. Building fingerprints. The target sequence copy number is calculated by dividing the target coverage by the genome coverage (top). This copy number is subsequently used to estimate the haplotype occurrences in the bacterial genome (bottom)

Strain	Genome length	Genome no of bases	Target length	Target no of bases	Genome coverage	Target Coverage	*16S rRNA* copy number	Rounded *16S rRNA* copy number
**KB_12**	6,249,593	343,125,462	1,511	328,250	54.90365	217.2402	3.956754	4

Haplotype	Target length	No of pseudoaligned reads	Ratio	rounded ratio	*16S rRNA* copy number	Occurrence
**H1**	1,511	1,256.7	3.459172	3	4	3
**H2**	1,511	363.295	1	1	4	1

Appendix 2 – SynCom data library preparation

To generate data for the SynCom reconstitution experiment, the *16S rRNA* V3-V4 and V5-V7 regions were subjected to high throughput two-step barcoding, purified using the AMPure XP Reagent for PCR purification (Beckman Coulter), purified for plant organelle-derived *16S rRNA* V5-V7 sequences, pooled and sequenced using a Novaseq 6000 PE250 sequencing run (2 x 250bp) (Table 1 and 2). The description of the shotgun metagenome-sequenced dataset can be found in (Selten et al., 2024).

Table 1. PCR1 primers for *16S rRNA* V3-V4 and V5-V7 sequencing (top) and thermocycler settings of PCR 1 (bottom).

Primer name	Oligo sequence
***16S rRNA* V5-V7 F**	TCGTCGGCAGCGTCAGATGTGTATAAGAGACAGAACMGGATTAGATACCCKG
***16S rRNA* V5-V7 R**	GTCTCGTGGGCTCGGAGATGTGTATAAGAGACAGACGTCATCCCCACCTTCC
***16S rRNA* V3-V4 F**	TCGTCGGCAGCGTCAGATGTGTATAAGAGACAGCCTACGGGNGGCWGCAG
***16S rRNA* V3-V4 R**	GTCTCGTGGGCTCGGAGATGTGTATAAGAGACAGGACTACHVGGGTATCTAATCC

PCR1 settings	T (°C)	Time	
**First denaturation**	95	2 min	
**Denaturation**	95	30 s	x30 cycles
**Annealing**	55	30 s	
**Elongation**	72	45 s	
**Final elongation**	72	10 min	
**End**	12	∞	

Table 2. PCR2 primers for V3-V4 and V5-V7 *16S rRNA* sequencing and PCR2 thermocycler settings. This step consisted of one universal forward primer and barcoded reverse primers for amplification

	Primer name	Oligo sequence
V3-V4	**UDP0200-i5**	AATGATACGGCGACCACCGAGATCTACACAATGTATTGCTCGTCGGCAGCGTC
V5-V7	**UDP0201-i5**	AATGATACGGCGACCACCGAGATCTACACGATCTCTGGATCGTCGGCAGCGTC
	UDP0289V2-i7	CAAGCAGAAGACGGCATACGAGATGCTACTATCTGTCTCGTGGGCTCGG
	UDP0289-i7	CAAGCAGAAGACGGCATACGAGATGGAATTGTTCGTCTCGTGGGCTCGG
	UDP0290V2-i7	CAAGCAGAAGACGGCATACGAGATGTCTTCTAATGTCTCGTGGGCTCGG
	UDP0290-i7	CAAGCAGAAGACGGCATACGAGATCCGGACCACAGTCTCGTGGGCTCGG
	UDP0291V2-i7	CAAGCAGAAGACGGCATACGAGATATGTGCGAGCGTCTCGTGGGCTCGG
	UDP0291-i7	CAAGCAGAAGACGGCATACGAGATGACTTAGAAGGTCTCGTGGGCTCGG
	UDP0292-i7	CAAGCAGAAGACGGCATACGAGATTGGCAATATTGTCTCGTGGGCTCGG
	UDP0293-i7	CAAGCAGAAGACGGCATACGAGATGAATGCACGAGTCTCGTGGGCTCGG
	UDP0294-i7	CAAGCAGAAGACGGCATACGAGATCGTGTATCTTGTCTCGTGGGCTCGG
	UDP0295-i7	CAAGCAGAAGACGGCATACGAGATATTCATTGCAGTCTCGTGGGCTCGG
	UDP0296-i7	CAAGCAGAAGACGGCATACGAGATTCCTTCATAGGTCTCGTGGGCTCGG
	UDP0297-i7	CAAGCAGAAGACGGCATACGAGATTCTAGTCTTCGTCTCGTGGGCTCGG
	UDP0298-i7	CAAGCAGAAGACGGCATACGAGATCTCGACTCCTGTCTCGTGGGCTCGG
	UDP0299-i7	CAAGCAGAAGACGGCATACGAGATAGTGAGTGAAGTCTCGTGGGCTCGG

	UDP0300-i7	CAAGCAGAAGACGGCATACGAGATGAAGCGGACCGTCTCGTGGGCTCGG
	UDP0301V2-i7	CAAGCAGAAGACGGCATACGAGATCAAGCCACTAGTCTCGTGGGCTCGG
	UDP0301-i7	CAAGCAGAAGACGGCATACGAGATGCTCTCGTTGGTCTCGTGGGCTCGG
	UDP0302-i7	CAAGCAGAAGACGGCATACGAGATGGACCTCAATGTCTCGTGGGCTCGG
	UDP0303-i7	CAAGCAGAAGACGGCATACGAGATGAGTCTCTCCGTCTCGTGGGCTCGG
	UDP0304-i7	CAAGCAGAAGACGGCATACGAGATAACGGAGCGGTCTCGTGGGCTCGG
	UDP0305-i7	CAAGCAGAAGACGGCATACGAGATTGTGATGTATGTCTCGTGGGCTCGG
	UDP0306-i7	CAAGCAGAAGACGGCATACGAGATAACATACCTAGTCTCGTGGGCTCGG
	UDP0307-i7	CAAGCAGAAGACGGCATACGAGATGTGCTAGGTGGTCTCGTGGGCTCGG
	UDP0308-i7	CAAGCAGAAGACGGCATACGAGATCATACTTGAAGTCTCGTGGGCTCGG
	UDP0309-i7	CAAGCAGAAGACGGCATACGAGATCTTGTCTTAAGTCTCGTGGGCTCGG
	UDP0310-i7	CAAGCAGAAGACGGCATACGAGATAAGAGAGGTGGTCTCGTGGGCTCGG
	UDP0311-i7	CAAGCAGAAGACGGCATACGAGATTGCACGAGAAGTCTCGTGGGCTCGG
	UDP0312-i7	CAAGCAGAAGACGGCATACGAGATACTTCCTAGCGTCTCGTGGGCTCGG
	UDP0313-i7	CAAGCAGAAGACGGCATACGAGATGTGCTATTAAGTCTCGTGGGCTCGG
	UDP0314-i7	CAAGCAGAAGACGGCATACGAGATAGCGTGAATGGTCTCGTGGGCTCGG
	UDP0315-i7	CAAGCAGAAGACGGCATACGAGATCCTTAGTGCCGTCTCGTGGGCTCGG
	UDP0316-i7	CAAGCAGAAGACGGCATACGAGATTGTACCGAATGTCTCGTGGGCTCGG
	UDP0317-i7	CAAGCAGAAGACGGCATACGAGATGGAGATTAGTGTCTCGTGGGCTCGG
	UDP0318-i7	CAAGCAGAAGACGGCATACGAGATTACTAACACAGTCTCGTGGGCTCGG
	UDP0319-i7	CAAGCAGAAGACGGCATACGAGATTAGGTCGTTGGTCTCGTGGGCTCGG
	UDP0320-i7	CAAGCAGAAGACGGCATACGAGATATGCCGACCGGTCTCGTGGGCTCGG
	UDP0321-i7	CAAGCAGAAGACGGCATACGAGATCTAGCGTCGAGTCTCGTGGGCTCGG
	UDP0322-i7	CAAGCAGAAGACGGCATACGAGATTGCCTACGAGGTCTCGTGGGCTCGG
	UDP0323-i7	CAAGCAGAAGACGGCATACGAGATACTAGAACTTGTCTCGTGGGCTCGG
	UDP0324-i7	CAAGCAGAAGACGGCATACGAGATCACCTCTTGGGTCTCGTGGGCTCGG
	UDP0325-i7	CAAGCAGAAGACGGCATACGAGATAAGCAGATATGTCTCGTGGGCTCGG
	UDP0326-i7	CAAGCAGAAGACGGCATACGAGATGCCAGATCCAGTCTCGTGGGCTCGG
	UDP0327-i7	CAAGCAGAAGACGGCATACGAGATTTGGATTCAAGTCTCGTGGGCTCGG

PCR 2 settings	T°C	Time	
**First denaturation**	95	2 min	
**Denaturation**	95	30 s	x10 cycles
**Annealing**	55	30 s	
**Elongation**	72	45 s	
**Final elongation**	72	10 min	
**End**	12	∞	

Appendix 3 – Contamination, heterogeneity, and GC content affects determination of *16S rRNA* copy number

Several of the 447 plant root bacterial isolates are indicated by exceptionally high *16S rRNA* copy numbers, with more than twenty *16S rRNA* sequences found in these genomes (Manuscript: Figure 3A and C). Interestingly, these high *16S rRNA* copy numbers are also observed in a few complete NCBI genomes (Manuscript: Figure S3A and S4). To investigate whether these high *16S rRNA* copy numbers are real and not technical artifacts, we correlated the *16S rRNA* copy number with the level of contamination and heterogeneity in the genome assemblies that we derived from CheckM (version 1.1.3) (Parks et al., 2015). Contamination and heterogeneity occur when single-copy marker genes are found more than once, either due to contamination from another bacterium (contamination) or from a duplication event (heterogeneity). In addition, a contamination from a phylogenetically closely related strain is often recognized as heterogeneity as well. Evidently, we discovered a weak correlation between the *16S rRNA* copy number and contamination level and no correlation with the heterogeneity level (Appendix Figure 1A). Conclusively, *16S rRNA* copies from contaminating sequences does not explain the exceptionally high *16S rRNA* copy numbers, though may play role for a couple of genomes.

Another explanation for the exceptionally high number of *16S rRNA* copies could be related to the GC content. Whole-genome sequencing of bacterial strains does not result in uniform read distribution or coverage across the entire genome, as GC-rich regions can experience higher coverage as compared to GC-poor regions (Chen et al., 2013; Tyler et al., 2016; Gunasekera et al., 2021). To investigate this, we correlated the GC content of the *16S rRNA* fingerprints to the *16S rRNA* copy number (Figure 1B). We also compared the GC content of the *16S rRNA* sequence from the complete NCBI genomes with their *16S rRNA* copy number to assess to what extent GC content correlates with overestimation of *16S rRNA* copy number (Appendix Figure 1C). Indeed, we found a significant positive correlation between the GC content of the *16S rRNA* fingerprint or sequence and the *16S rRNA* copy number in both datasets (Appendix Figure 1B and C). By comparing the correlation slopes between the two datasets we can estimate the extent to which SyFi may overestimate the *16S rRNA* copy number compared to copy numbers of closed genomes, assuming that fully assembled genomes did not lose any *16S rRNA* copies. The difference in correlation slopes indicates how SyFi might be extensively overestimating the *16S rRNA* copy number at high GC contents in the bacterial genome (Figure 1D black line). This observation, however, may also potentially differ between different bacterial taxonomic groups, corroborating the need for a phylogenetic marker gene database in future implementations of SyFi.

Conclusively, contamination may contribute to high, biologically infeasible *16S rRNA* copy numbers. In spite of this finding, a high GC content seems mainly responsible for *16S rRNA* copy number overestimation. In future versions, a GC content normalization of the *16S rRNA* copy number may be implemented in SyFi to account for this overestimation.

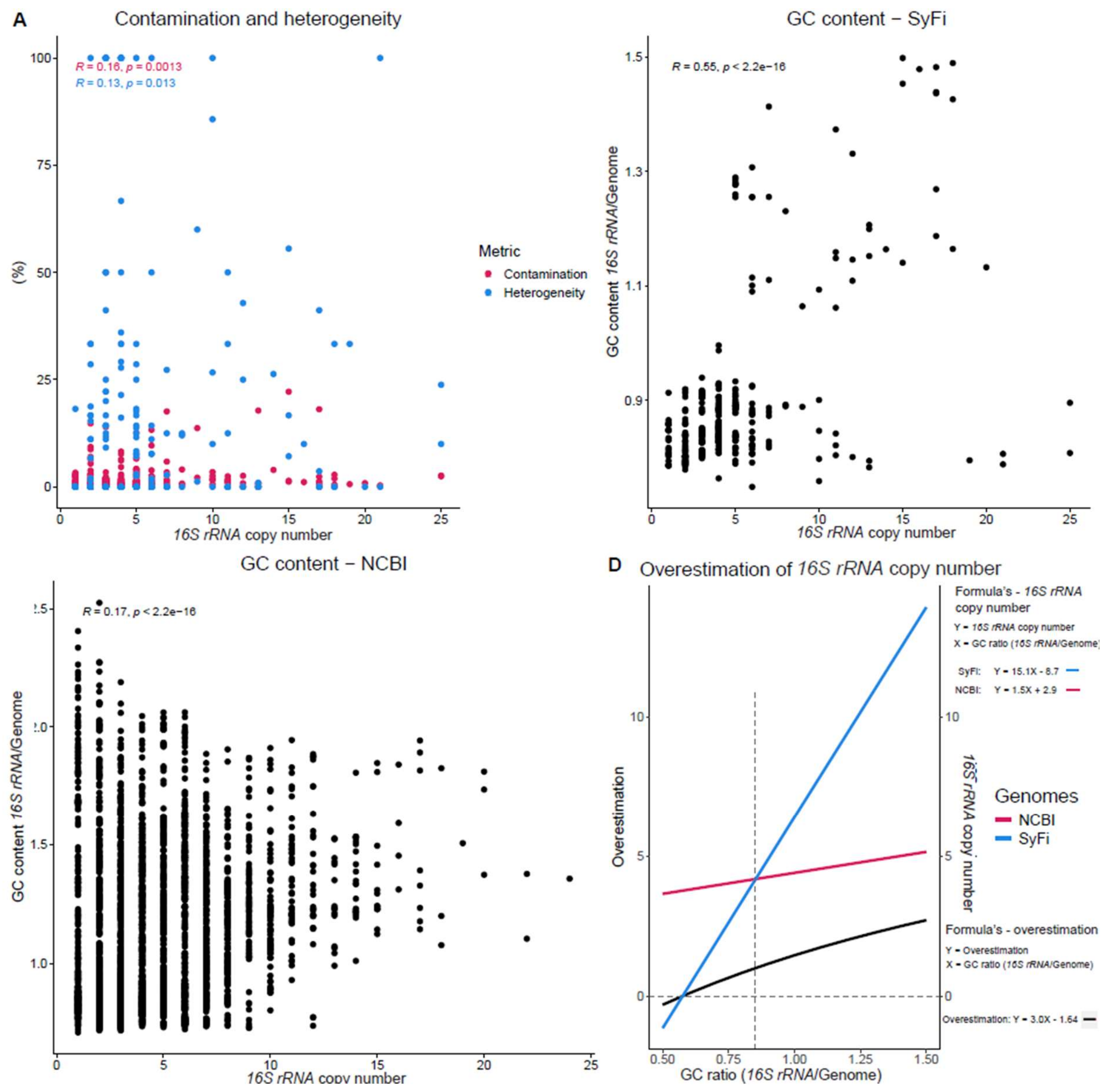

Appendix Figure 1. The effect of contamination, heterogeneity, and GC content on the *16S rRNA* copy. Contamination and heterogeneity (A) (y-axis) in the genome assemblies correlate weakly but significantly with the *16S rRNA* copy number (x-axis). A significant positive correlation exists between proportion of GC content in the *16S rRNA* sequence as compared to the genome (B) (y-axis) and the *16S rRNA* copy number (x-axis). The *16S rRNA* copy number in complete NCBI genomes is also affected by the difference in GC content between the *16S rRNA* sequence and the genome (C). The correlation between *16S rRNA* copy number and *16S rRNA*-genome GC content among the genomes used in SyFi as well as the closed NCBI genomes is compared to each other to illustrate the overestimation of *16S rRNA* copy number (D).

References

Altschul, S. F., Gish, W., Miller, W., Myers, E. W., & Lipman, D. J. (1990). Basic local alignment search tool. *Journal of Molecular Biology*, **215**, 403–410.

Bankevich, A., Nurk, S., Antipov, D., Gurevich, A. A., Dvorkin, M., Kulikov, A. S., Lesin, V. M., Nikolenko, S. I., Pham, S., Prjibelski, A. D., Pyshkin, A. V., Sirotkin, A. V., Vyahhi, N., Tesler, G., Alekseyev, M. A., & Pevzner, P. A. (2012). SPAdes: a new genome assembly algorithm and its applications to single-cell sequencing. *Journal of Computational Biology*, **19**, 455–477.

Bray, N. L., Pimentel, H., Melsted, P., & Pachter, L. (2016). Near-optimal probabilistic RNA-seq quantification. *Nature Biotechnology*, **34**, 525–527.

Chen, Y.-C., Liu, T., Yu, C.-H., Chiang, T.-Y., & Hwang, C.-C. (2013). Effects of GC bias in next-generation-sequencing data on de novo genome assembly. *PLOS ONE*, **8**, e62856.

Gunasekera, S., Abraham, S., Stegger, M., Pang, S., Wang, P., Sahibzada, S., & O'Dea, M. (2021). Evaluating coverage bias in next-generation sequencing of *Escherichia coli*. *PLOS ONE*, **16**, e0253440.

Li, H., & Durbin, R. (2009). Fast and accurate short read alignment with Burrows–Wheeler transform. *Bioinformatics*, **25**, 1754–1760.

Li, H., Handsaker, B., Wysoker, A., Fennell, T., Ruan, J., Homer, N., Marth, G., Abecasis, G., & Durbin, R. (2009). The sequence alignment/map format and SAMtools. *Bioinformatics*, **25**, 2078–2079.

Martin, M., Patterson, M., Garg, S., O Fischer, S., Pisanti, N., Klau, G. W., Schöenhuth, A., & Marschall, T. (2016). WhatsHap: fast and accurate read-based phasing. *BioRxiv*, 085050.

McKenna, A., Hanna, M., Banks, E., Sivachenko, A., Cibulskis, K., Kernytsky, A., Garimella, K., Altshuler, D., Gabriel, S., Daly, M., & DePristo, M. A. (2010). The Genome Analysis Toolkit: a MapReduce framework for analyzing next-generation DNA sequencing data. *Genome Research*, **20**, 1297–1303.

Parks, D. H., Imelfort, M., Skennerton, C. T., Hugenholtz, P., & Tyson, G. W. (2015). CheckM: assessing the quality of microbial genomes recovered from isolates, single cells, and metagenomes. *Genome Research*, **25**, 1043–1055.

Selten, G., Lamouche, F., Gómez-Repollés, A., Blahovska, Z., Kelly, S., de Jonge, R., & Radutoiu, S. (2024). Functional capacities drive recruitment of bacteria into plant root microbiota. *BioRxiv*, 2024–2028.

Tyler, A. D., Christianson, S., Knox, N. C., Mabon, P., Wolfe, J., Van Domselaar, G., Graham, M. R., & Sharma, M. K. (2016). Comparison of sample preparation methods used for the next-generation sequencing of *Mycobacterium tuberculosis*. *PLOS ONE*, **11**, e0148676.
